# Reference Gene-Assisted LAMP–LFD for Sensitive and Specific Detection of Soy DNA as a Marker for Allergen Presence in Complex Food Products

**DOI:** 10.1021/acs.jafc.5c01463

**Published:** 2025-04-24

**Authors:** Qiaofeng Li, Marleen M. Voorhuijzen-Harink, Dianpeng Han, Bas J. Fronen, Richard van Hoof, Ming Chen, Zhouping Wang, Toine F. H. Bovee, Zhixian Gao, Gert IJ. Salentijn

**Affiliations:** † Department of Clinical Laboratory Medicine, Southwest Hospital, Third Military Medical University (Army Medical University), 30 Gaotanyan, Shapingba District, Chongqing 400038, China; ‡ Wageningen Food Safety Research, Wageningen University & Research, P.O. Box 230, Wageningen 6700 AE, The Netherlands; § Tianjin Key Laboratory of Risk Assessment and Control Technology for Environment and Food Safety, Military Medical Sciences Academy, Tianjin 300050, China; ∥ State Key Laboratory of Food Science and Technology, Jiangnan University, Wuxi 214122, China; ⊥ Laboratory of Organic Chemistry, Wageningen University, Wageningen 6708 WE, The Netherlands

**Keywords:** soy allergen, loop-mediated isothermal amplification, lateral flow device, real food samples, rapid detection

## Abstract

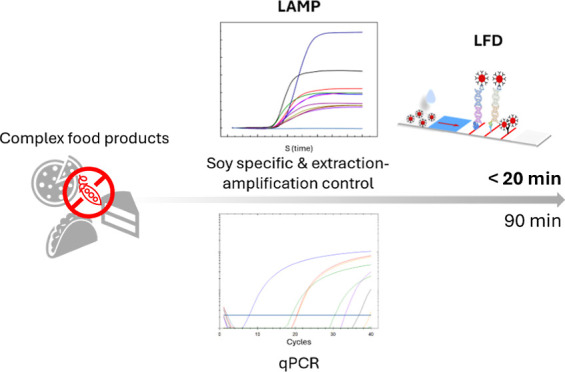

Sensitive, selective screening for allergenic ingredients with internal control is crucial to identify food adulteration and remove allergens from the food chain. Here, loop-mediated isothermal amplification combined with a lateral flow device (LAMP–LFD) was developed for the fast and easy detection of soy DNA. The integrated quality controls included a regular control line to ensure proper implementation (extraction and amplification control) and a second LAMP–LFD assay for the cytochrome oxidase gene, which is a housekeeping gene in plants. The developed LAMP assay showed a limit of detection of only 5 pg DNA input per reaction, for both pure soy and spiked food samples. The test was subsequently implemented for the examination of 32 real food products with different compositions and declared soy contents, and benchmarked against qPCR. Then, this system was combined with a digital cube reader, allowing direct interpretation of the test results, facilitating the point-of-need applicability.

## 1 Introduction

Soybean (*Glycine max*) is a common ingredient in food products. Soy allergy, however, is one of the most common food allergies affecting millions of people globally,^1^,^2^ and can lead to severe shock and even death. Unfortunately, there is no effective way to mitigate food allergies, except fully avoiding exposure to allergenic ingredients, which requires rigor and constant vigilance. Food labeling laws mandate that the manufacturers must declare intentionally added soy and other allergens in food products.^3^ However, it remains extremely difficult to completely avoid their ingestion, due to undeclared allergenic ingredients, mislabeling, ambiguous preventive labeling, or unintentional cross-contamination of allergens in the food chain,^4^.^5^ For food producers (factories or restaurants), it is important to detect any unwanted allergen presence, e.g., on a production line or in food products, including raw materials and food on sale, to avoid financial and reputation loss.^6^ It is therefore of substantial added value to have access to sensitive, accurate, rapid, and reliable methods to determine soy in food products, ideally on the spot. This would have the potential to reduce the burden on food producers and inspectors, and facilitate consumers in choosing their food in an evidence-based manner.

Currently, the most common approaches for allergen detection are immunochemical, nucleic-acid–based, and chromatography-based methods,^2^.^7^ Although being specific and sensitive, the utilization of these methodologies in the on-site analysis of samples can be limited due to the associated workload and costs. Hence, it is imperative to develop methods that can perform highly sensitive and specific yet cost-effective measurements. In addition, the accurate detection of trace amounts of allergens is complicated by the impact of diverse food processing and food matrices. Tobridge the gap between the lab-based and on-site detection, isothermal DNA amplification techniques, such as loop-mediated isothermal amplification (LAMP), have been proposed as viable alternatives.^8^ LAMP has been demonstrated to have advantages over PCR in terms of ease-of-operation and speed of detection.^9^ Part of those advantages come from the use of 4 main primers (inner and outer primers) and loop primers to accelerate the amplification, and the fact that there is no need for thermocycling, but rather a heating block at a constant temperature in the range of 60–65 °C. Moreover, the readout of amplified DNA products can be easily achieved by visual detection with the naked eye or a reader by the addition of detection probes or an intercalating dye.^10^

Additionally, detection by truly portable systems, such as lateral flow devices (LFD) or microfluidic chips, has demonstrated the potential for on-site testing and rapid investigation of incidents,^11^.^12^ Recent work has demonstrated LAMP assays for allergen testing, highlighting how the combination of LAMP and LFD can play an important role in testing food production lines.^13^ Nevertheless, one key element of such assays, especially if intended for on-site testing, is the risk of false outcomes, and especially false negatives for trace concentrations are of concern as those might trigger allergic reactions. As an example, improper DNA extraction could lead to such false negative outcomes, and with a regular LAMP–LFD such an event would remain unnoticed—that is, until the food is consumed. Moreover, a limitation of the LFD is that, although users can obtain results, the information cannot be stored, analyzed, and shared easily. The problem of data traceability can be addressed using smartphones for detection with the camera, and data storage/transmission. Finally, “food” is an immense category of diverse matrices, with widely varying composition, and as such, it is critical to validate any method properly in a wide variety of food matrices. Therefore, new methods should be developed that acknowledge and address the aforementioned limitations.

In this work, four aspects were explored to advance the current LAMP methods and to ensure reliable and accurate results in soy determination in food: (1) a LAMP assay was developed targeting a specific soy gene and the assay conditions (primer sets, temperature, and the ratio of primers) were optimized; (2) a second target, namely, the cytochrome oxidase (Cox) gene, was implemented for LAMP–LFD detection as a control for successful DNA extraction and amplification; (3) to check the broad applicability in food analysis, 32 representative complex foods were tested by the proposed assay and outcomes were benchmarked with qPCR; (4) to ensure traceability and shareability of test results, a digital cube reader was combined with the LAMP–LFD system to store, analyze, and share the data, making this a smart and evidence-based on-site applicable detection platform.

## 2 Materials and Methods

### 2.1 Experimental Design

**Figure 1:**
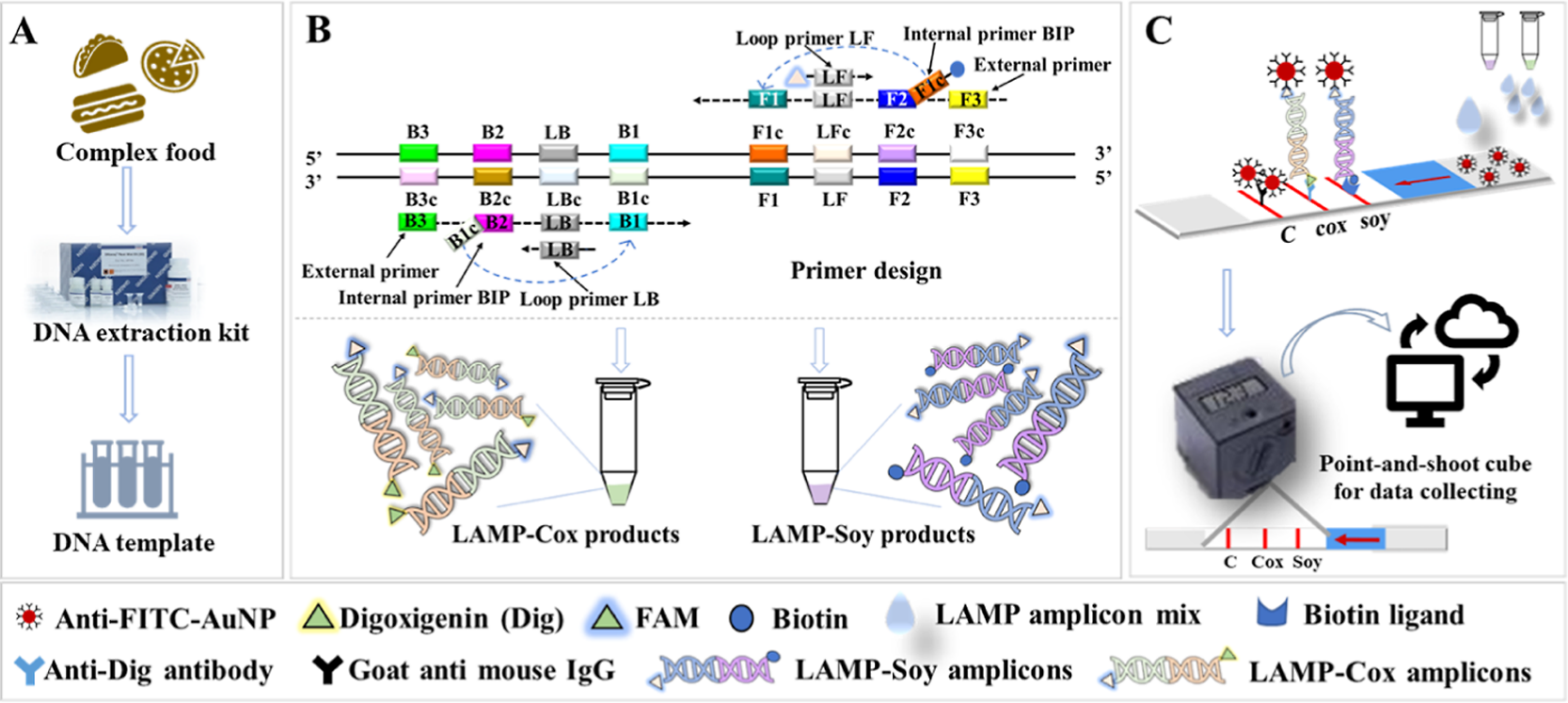
Workflow of detecting soy allergens based on duplex LAMP–LFD. (A) DNA extraction; (B) development of the LAMP assays; (C) the portable duplex LAMP–LFD and digital readout system.

The development of this method is schematically depicted in Figure 1. Genomic DNA of different food matrices was obtained using a commercial plant DNA extraction kit (Figure 1A). LAMP primers were designed or taken from literature for soy,^12^ and primers for the housekeeping Cox gene were taken from literature (Figure 1B, and Section 2.5).^14^ The best performing soy LAMP assay was selected and optimized (soy assay, Section 2.6). Limit of detection (LOD) was determined for the soy and Cox assays (Section 2.7). For LFD interpretation, the forward inner primer (FIP) of the soy and the Cox assays were labeled with biotin and digoxin (Dig), respectively; the loop primers were labeled with fluorescein phosphoramidite (FAM) (Section 2.8). Gold particles modified with fluorescein isothiocyanate (FITC)-antibodies on the LFD can combine with the labeled soy and Cox products. After the LAMP reaction, the labeled primers in the products were trapped on the test/control line and the result was interpreted either by eye or a digital readout cube (Figure 1C, and Section 2.9). For qualitative or semiquantitative detection, a point-and-shoot lateral flow reader connected to a laptop was used, after which the analyzed data were stored and could be shared.

### 2.2 Reagents and Consumables

Isothermal Mastermix (ISO-004, Optigene) or Diagenode Universal Mastermix (DMML-D2-D600, Diagenode Diagnostics) was used for LAMP and qPCR, respectively. Disposable sterile sampling spoons were used as tools for sample processing, and all solutions were made with the nuclease-free water purchased from the Thermo Fisher Scientific. A Qiagen DNeasy Plant Mini Kit was used to extract the genomic DNA (Qiagen, Hilden, Germany). A Milenia HybriDetect 2T kit was used for the lateral flow testing (Milenia, Germany). All the oligonucleotides’ sequences (Tables S1 and S2) were synthesized by Integrated DNA Technologies (IDT, USA). The digital lateral flow reader (Cube Reader) was used for the on-site applicable readout of the LAMP–LFD assay (Chembio Diagnostic, Germany).

### 2.3 Samples and Sample Preparation

Various well-described plant species were available at Wageningen Food Safety Research and were used to evaluate the specificity (Table S3). In addition, a total of 32 commercial food samples were sourced from the local supermarkets in The Netherlands. The characteristics (soy contents and three macronutrients) of the analyzed samples and their classification based on texture are shown in Table S4. Prior to DNA extraction, the solid samples were ground using a mortar and pestle. The half-solid samples such as vegetable salads were cut with scissors, and partially mixed pieces were taken as samples. The viscous liquid and emulsions were mixed before use. The liquid samples were directly used. All the samples were taken using a disposable sterile sampling spoon. The above processed samples were stored at −20 °C until further use.

Plant-based pure DNAs were extracted using in-house available extraction methods at Wageningen Food Safety Research, and the quality and quantity of the obtained genomic DNA were determined with a nanodrop. Total genomic DNA from commercial food products was extracted with the Qiagen DNeasy Plant Mini Kit (Qiagen, Hilden, Germany) according to the manufacturer’s instructions: 100 mg (wet weight) or 20 mg (dried) of crushed food samples, 400 μL of buffer AP1, and 4 μL of RNase solution (100 mg/mL) were mixed in a 1.5 mL microcentrifuge tube and incubated at 65 °C for 10 min. Then 130 μL of buffer P3 was added to the lysate and this was incubated for another 5 min. After the centrifugation at 14,000 rpm for 5 min, the lysate was pipetted into the QIAshredder Mini spin column with a 2 mL collection tube, followed by centrifugation at 14,000 rpm for 2 min. Subsequently, the flow-through part was transferred to a new tube and mixed with 1.5 times volumes of buffer AW1. Then, the mixture was transferred into a DNeasy Mini spin column with a 2 mL collection tube and centrifuged for 1 min at 8000 rpm. The flow-through and collection tube were discarded and replaced by a new 2 mL collection tube. Then, 500 μL of Buffer AW2 was added into the column, and this mixture was centrifuged for 1 min at 8000 rpm. After another centrifugation for 2 min at 14,000 rpm, the membrane in the column was dried. Finally, the DNeasy Mini spin column was placed in a 2 mL microcentrifuge tube, with addition of a 100 μL of Buffer AE on the DNeasy membrane and after 5 min incubation, the final DNA solution was obtained by centrifugation for 1 min at 10,000 rpm in the collection tube. Samples and extracted DNA were stored at −20 °C until use.

### 2.4 qPCR Assay

qPCR was used as benchmark for assay evaluation. The qPCR primer/probe sequences for the soy-specific gene and the plant gene (namely, actin gene as extraction and amplification control) are shown in Table S1. The qPCR was carried out with the Biorad CFX96 real-time system thermal cycler with automatic baseline settings. The reaction mixture contained 12.5 μL of Diagenode 1 × MM; 400 nM forward primer; 400 nM reverse primer; 200 nM probe primer; and 5 μL of template DNA and sterile water that was added to a final volume of 25 μL. The thermal cycling protocol for qPCR was the Decontamination UNG digestion step at 50 °C for 2 min, followed by a denaturation step for 10 min at 95 °C, and 45 cycles, each of: 15 s at 95 °C for denaturation, 60 s at 60 °C for annealing and extending.

### 2.5 LAMP Primers Design

The glycine max mitochondrion genome sequence (Genbank NC_020455.1: c205712–205230) of soy was used for the design of two LAMP primers sets via a primer design tool on the website https://lamp.neb.com/#!/ (Supporting Information, Table S2 for detailed information); one soy primer set was taken from work by Allgöwer et al.^12^ In addition, a LAMP assay developed by Tomlinson et al. detecting the mitochondrial universal Cox gene was implemented with minor modification to the detection conditions in order to achieve higher detection sensitivity^14^ and to confirm the success of DNA extraction and amplification.

### 2.6 LAMP Assay Development and Optimization

The final LAMP reactions were performed in a total reaction volume of 25 μL containing 0.2 μM of F3 and B3 each, 1.2 μM (soy gene)/1.6 μM (Cox gene) of FIP and BIP each, 0.4 μM of Loop F and Loop B primer each, 1× isothermal Mastermix (Optigene ISO-004), and 5 μL of extracted DNA (10 ng/μL) as template, or sterile water as no-template control (NTC). Finally, the LAMP process for both gene systems was monitored by fluorescence detection via the CFX96 real-time system Thermal Cycler (Biorad).

In the initial development stage, the best performing LAMP assay was determined by comparing different primer sets (set 1, set 2, set 3), temperatures (62, 63, 64, 65 °C), and ratios of inner and outer primers (inner/outer primer at 2:1, 4:1, 6:1, 8:1, 10:1) in the reaction system.

### 2.7 Performance Evaluation of the Developed LAMP Assay

To examine the specificity of the developed LAMP-soy assay, 18 plant species (Table S3) that are commonly used in food products were tested. Next, to determine the sensitivity of this method, the developed LAMP assay was performed using serial dilutions of soy DNA templates ranging from 5 × 10^–5^ to 5 ng of soy DNA per LAMP reaction. The LOD was set as the lowest concentration at which all 20 tests were positive. In addition, to check the LOD in spiked samples, multiple low concentrations of soy DNA were spiked into two samples without a detectable amount of soy and two declared soy-free samples. The LOD was set as the lowest concentration at which all 10 replicates were positive.

### 2.8 LAMP–LFD Assay Development and Characterization

For the LAMP–LFD assay, soy-FIP and Cox-FIP were individually 5′ modified by biotin or Dig, respectively, and soy-Loop F and Cox-Loop F were both 5′ modified by FAM. The ready-to-use duplex LFD (Milenia HybriDetect 2T, Germany) based on FITC antibody-modified gold nanoparticles in the sample application area of the dipstick was used to detect the amplification products. The LFD was functionated with two test lines and a control line (Supporting Information, Figure S1). Test line 1 contained immobilized biotin-ligand, test line 2 immobilized anti-Dig antibody, and the C line immobilized goat-antimouse IgG. During the LFD development, LAMP-soy amplicons were captured by biotin-ligand immobilized on test line 1, LAMP-Cox amplicons were captured by Dig-antibodies immobilized on test line 2, and the free gold-labeled FITC antibodies were captured on the control line.

The amplification protocol of the LAMP–LFD was the same as the standard LAMP reaction, except that labeled primers were used to replace the previous FIP and Loop primers. To obtain the real-time amplification data, the LAMP reaction was monitored by a Genie II (OptiGene, Horsham, UK). Then, the LFD was used to detect amplification products. First, the amount of amplification products loaded on LFD was optimized: equal amounts of LAMP-soy and LAMP-Cox reaction mixtures (1, 3, 5, 7, or 9 μL) were mixed in a well of a microtiter plate. To determine the presence of the high dose hook effect, the amplification products were further diluted in this step to achieve a dilution ratio (v/v) of 4:100, 2:100, 1:100, 1:200, 1:400, 1:800, 1:1600 in the final 100 μL of LFD buffer. Finally, the LFDs were placed with the sample application area into the solution and incubated for 10 min in upright position. After removing the LFD from the assay solution, the results were interpreted immediately by eye or a digital reader.

For the sensitivity of the LAMP–LFD system, a serial dilution of pure soy DNA solution from 5 × 10^–5^ to 5 ng per LAMP reaction was tested in duplicate, and the LOD was defined as the lowest detectable concentration of the DNA template giving a positive result in 10 tests. For the LOD determination in spiked samples, the detection limit was defined as the lowest concentration of spiked soy DNA (in 10 tests) that was detected in two soy-free samples.

### 2.9 Digital Readout of LAMP–LFD Assay

The LFD can be read out visually, or by an automated digital lateral flow reader (Cube Reader; Chembio Diagnostic, Germany). For the last, the LFD was put into the cavity of a cassette, which was subsequently inserted into the Cube Reader. A radio frequency identification (RFID)-tag card with customized loading program for the detection of soy allergen was used. After identifying the assay with the card, the reading was started, and a positive or negative result appeared in the readout display several seconds later. To explore the possibility of semiquantitative detection using this readout method, different amounts of soy DNA (5 × 10^–5^ to 5 ng per LAMP reaction) were tested by the LAMP–LFD assay, and the gray value of each line on the LFD was analyzed by the digital Cube Reader. By connecting to a computer or smartphone using a cable, the results could be saved digitally and processed further.

### 2.10 Application to Commercial Food Samples

Thirty-two real food samples with different soy content were chosen and classified by their textural characteristics, namely, (1) hard solid, (2) gelatinous and soft-solid, (3) viscous liquid and emulsions, and (4) liquid samples (see Supporting Information Table S4). The samples were homogenized, and DNA was extracted as described in Section 2.3. Three samples with different soy content #1 (100% soy), #11 (50%), #19 (>1% soy) were first tested to check the applicability of the developed assays in real samples. After DNA extraction, LAMP reactions were carried out with 2 μL of extract of the three commercial food products. In the testing of commercial foods, the concentration of total extracted DNA is not determined, which also would not be done in an on-site testing scenario. Analysis was performed by monitoring the amplification curves and melting temperature, and subsequently the LAMP products were detected by LFD.

Next, all 32 samples were analyzed by using 2 μL of extract as the template. All the amplification products were detected by real-time measuring fluorescence with a PCR thermal cycler. DNA of each sample was extracted twice. The extracted DNA from the high soy content samples were analyzed in duplicate, and four tests were conducted for low soy content samples. Finally, 10 representative samples with high to low soy content were tested by LAMP, LAMP–LFD, and qPCR.

## 3 Results and Discussion

### 3.1 LAMP Assay Development and Optimization

To achieve highly sensitive detection with the LAMP-soy system, optimization was conducted to find the best soy primer set. Figure S2A (Supporting Information) confirmed three functional primer sets. The high variability of set 2 in amplification time under minor temperature shifts prompted its exclusion due to low robustness. Then, set 1 and set 3 were tested with varying soy DNA inputs at 62 and 65 °C (Supporting Information, Figure S2B), yielding comparable results. However, shorter target sizes are preferred for complex processed food products as DNA degradation is known to occur due to processing; therefore set 1 (target size 217 bp) was chosen over set 3 (target size 310 bp). Moreover, for LAMP-Cox, 65 °C was selected based on prior optimization studies,^14^ which is why set 1 and 65 °C were selected for further experimentation. Next, the impact of different ratios of inner and outer primers on the LAMP reaction was explored. When the ratios of inner and outer primers were 6 and 8, the LAMP reaction for both soy and Cox genes reached the fluorescence threshold with the greatest repeatability (RSDs < 5%) and highest efficiency (amplification times less than 8.5 min) (Supporting Information, Figure S2C). Therefore, these optimized conditions were implemented in the LAMP assays, i.e., set 1, at 65 °C and inner-outer primers ratio of 6.

### 3.2 Performance Evaluation of the LAMP Assays

To assess the specificity of the soy LAMP assay, 18 edible plant species were tested applying the optimized conditions. As shown in Table 1, none of the other pure plant DNA samples were detected. Only soy was specifically amplified by the soy LAMP assay (see Supporting Information Figure S3 for amplification and melting curves). At the same time, the presence of plant DNA was successfully confirmed by the detection of the Cox gene in all 18 plant species. It is worth highlighting that although white lupine, peanut, pea and kidney bean show genetic homology with soy (all belong to the *Fabaceae* family), nonspecific amplification did not appear, underlining good specificity of the soy assay. These results were confirmed by qPCR (Table 1).

**Table 1:** Specificity of the LAMP Soy Assay with qPCR as a Benchmark^a^

	LAMP		qPCR	
name	soy	Cox	soy-specific	plant
white mustard	–	+	–	+
pistachio	–	+	–	+
sesame	–	+	–	+
celeriac	–	+	–	+
spelt	–	+	–	+
common wheat	–	+	–	+
common purslane	–	+	–	+
pecan	–	+	–	+
Brazil nut	–	+	–	+
macadamia	–	+	–	+
walnut	–	+	–	+
hazelnut	–	+	–	+
celery	–	+	–	+
white lupine	–	+	–	+
peanut	–	+	–	+
kidney bean	–	+	–	+
pea	–	+	–	+
soybean	+	+	+	+
MQ	–	–	–	–

a MQ is negative control with Milli-Q water.

To determine the sensitivity of the LAMP assays under optimized conditions, pure soy DNA was tested, and the results were compared to those obtained by qPCR analysis (Table 2 and Figure S4, Supporting Information). Both the soy gene and Cox gene could be detected at 5 × 10^–3^ ng pure soy DNA per LAMP reaction in less than 15 min. Even amplification down to 5 × 10^–4^ ng pure soy was observed, but inconsistently. The detection sensitivity of LAMP was comparable to qPCR; however, qPCR detection takes more than 1 h. In conclusion, the similar outcomes of the LAMP and qPCR analysis of the 18 samples and the high sensitivity, rapidness, and ease of the LAMP assays demonstrate the applicability for the on-site detection of the soy allergen by LAMP.

**Table 2:**
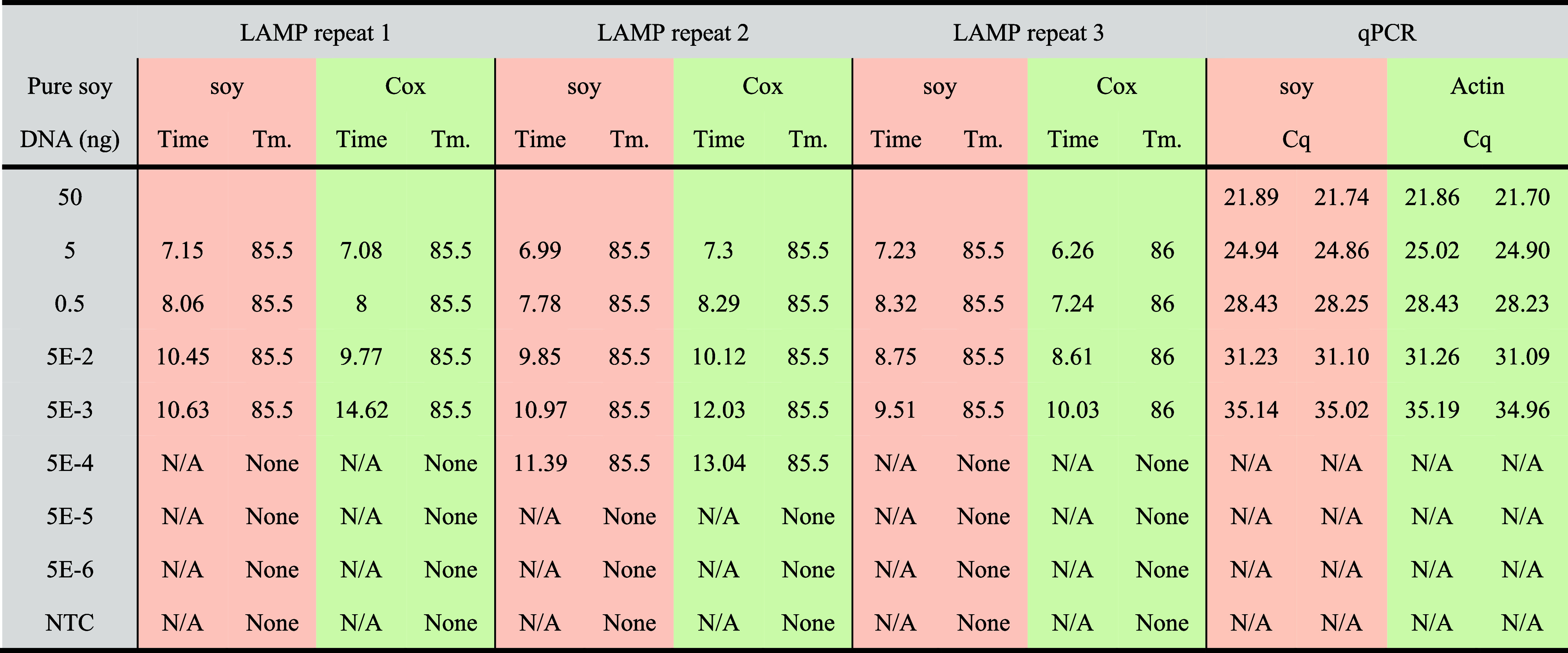
Sensitivity of LAMP and qPCR Assay for the Detection of Pure Soy DNA^a^

a Note: “Time” means amplification time (in minutes). “*T*_m_.” represents the melting temperature (in degrees C). “N/A” represents no detectable template. “None” represents no melting temperature was reported. “*C*_q_” represents the number of cycles at the intersection of PCR curve and threshold line. “NTC” represents no template control.

Table 3 illustrates the LOD determined for pure soy DNA and four spiked samples, which were declared not to contain soy ingredients (selected from 32 real food samples, Supporting Information, Table S4). The LAMP-soy reaction and LAMP-Cox reaction achieved LODs (20 out of 20 positive) as low as 5 × 10^–4^ and 5 × 10^–2^ ng input pure soy DNA, respectively. However, care must be taken regarding the LODs, especially for the soy assay. As can be observed in Table 2, here only one out of three replicates showed amplification at 5 × 10^–4^ ng pure soy DNA input, while as shown in Table 3, this same amount of input DNA, after fresh extraction, results in 20 out of 20 positive amplifications. As LAMP amplifies target DNA highly sensitively, small differences in input amount can have large effects, especially at very low input levels as the LOD. Besides, we also noticed that the LOD of the LAMP–Cox assay was higher compared to that of the LAMP–soy assay, indicating lower sensitivity. Similar sensitivity of the soy and Cox assays would be preferred, however, when care is taken with reading the results the Cox assay can still be applied. For example, if a sample only contains soy, positive amplification with only the soy assay would be sufficient to declare “soy detected”. If neither soy nor Cox shows positive amplification, the result is not trustworthy and needs to be repeated in case of a plant-based sample. On the contrary, if a sample would contain soy at a low percentage (>LOD) resulting in no amplification, and other plant species at higher levels resulting in positive Cox amplification as more targets are present, this would lead to the false assumption of correct performance. Therefore, consideration of the composition of the sample when reading the results is important. In the LAMP–Cox reaction, all the 32 food products were tested positive at the 5 × 10^–4^ ng spike level, and this can be attributed to the food products containing other plant materials besides the added soy (see also Table 3). For the determination of the soy LOD in four spiked samples, all materials with spiked soy DNA at 5 × 10^–3^ ng per LAMP-soy reaction were tested positive within 11 min. The reduced detectability in spiked samples compared to that of pure soy DNA indicates an inhibitory effect of the amplification in the LAMP–soy reaction.^15^

**Table 3:** LOD of Both LAMP Assays with Pure Soy DNA and Spiked Samples

LOD test	Cox gene test					soy gene test	
DNA/LAMP (ng)	5 × 10^–5^	5 × 10^–4^	5 × 10^–3^	5 × 10^–2^	5 × 10^–5^	5 × 10^–4^	5 × 10^–3^
pure soy DNA	13 (20)^a^	12 (20)	18 (20)	**20 (20)**	19 (20)	**20 (20)**	
spiked veggie soup (#24)	8 (10)	**10** (**10)**	10 (10)		9 (10)	8 (10)	**10 (10)**
spiked cookie 2 (#26)	9 (10)	**10 (10)**	10 (10)		8 (10)	8 (10)	**10 (10)**
spiked salad 3 (#28)		**10 (10)**	10 (10)			5 (10)	**10 (10)**
spiked coco milk (#31)		**10 (10)**	10 (10)			7 (10)	**10 (10)**

a The number of positive reactions in all repeated tests, for example, 13 (20) represents there were 13 positive reactions in 20 repeated tests.

### 3.3 Establishment of Duplex LAMP–LFD Assay

The use of an LFD for LAMP readout allows the direct visual inspection of the results and is therefore highly suitable for on-site detection. The high dose hook effect, which is mainly known to occur in sandwich-format LFDs^16^ has been reported for amplicon detection with LFD in PCR, and thus we investigated the influence on our LAMP–LFD system. In order to investigate this phenomenon, the amount of amplificons loaded on the LFD was first optimized. The LAMP assays developed in this study are governed by 6 primers and the high displacement activity of the Bst DNA polymerase, generating exponentially increasing amounts of DNA.^9^ Eventually, the excess of products that can bind both the LFD membrane and the gold-labeled antibodies will limit sandwich formation on the T-lines (as the chances of binding both become smaller with a larger excess of product). Indeed, as is shown in Supporting Information, Figure S5, the hook effect was also clearly observed visually, as well as by the quantitative analysis using ImageJ software, demonstrated by a decrease of the color intensity of the T line while increasing the amount of the LAMP reaction products. Since the color intensity with only 1 μL of input of LAMP amplification products diluted with 50 μL of running buffer (50 times diluted) already resulted in a very intense color of the T line, additional experiments were carried out using a higher dilution of the amplification products (100, 200, 400, 800 times diluted). By increasing the dilution ratio gradually, both the colored control and test lines became more intense (dilution up to 200 times), after which they gradually decreased again. Therefore, a 200 times dilution of the amplified products was used in subsequent experiments.

**Figure 2:**
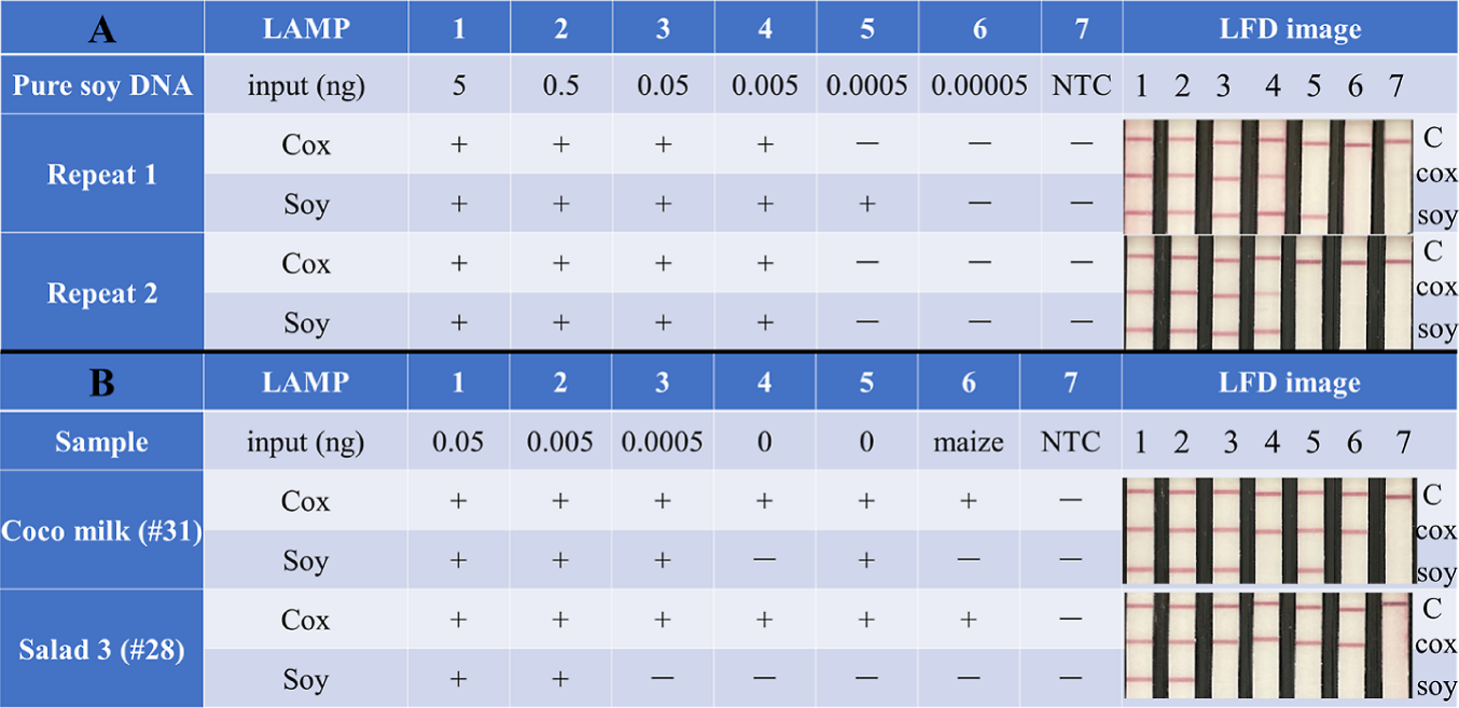
Performance of the LAMP and LAMP–LFD system. The sensitivity in (A) pure soy DNA and (B) spiked coco milk and salad samples. The results of the LAMP reaction as measured in Genie II are shown on the left side, indicated by a positive (+) or negative (−). A 200-fold dilution of the LAMP reaction product was also analyzed by LFD and the LFD images are included on the right side. NTC means no template control.

To assess the sensitivity of the LAMP–LFD system, different concentrations of pure soy DNA were amplified by LAMP and then detected by LFD. Figure 2 demonstrates that both the soy and Cox gene-based LAMP assays resulted in a positive amplification at the 5 × 10^–3^ ng DNA input in the LAMP reaction, and these could be detected by LAMP–LFD as well. Moreover, to check the LAMP–LFD performance in matrix, two soy-free samples were chosen for spiking with soy. As expected, the LAMP–Cox gene system tested positive for all samples, due to the presence of plant material. One nonspiked coco milk (sample #31) extraction resulted in amplification by the LAMP-soy gene system, but the duplicate analysis was negative, as was the qPCR control and LAMP-fluorescent readout assay in Table 3.

Next, the LOD of the LAMP–LFD was determined by 10 parallel tests using 0.05 and 0.005 ng of pure soy DNA (Figure S6). At 5 × 10^–2^ ng input, all samples showed clear color intensity on the three lines of the LFD. Although all C-lines and the 10 soy T-lines were positive at 5 × 10^–3^ ng input too, only 3 out of 10 Cox T-lines were obviously positive and 2 out of 10 showed slightly positive, which means that the LOD of LAMP–LFD assay was 5 × 10^–2^ ng per reaction.

### 3.4 Application to Commercial Food Samples

**Figure 3:**
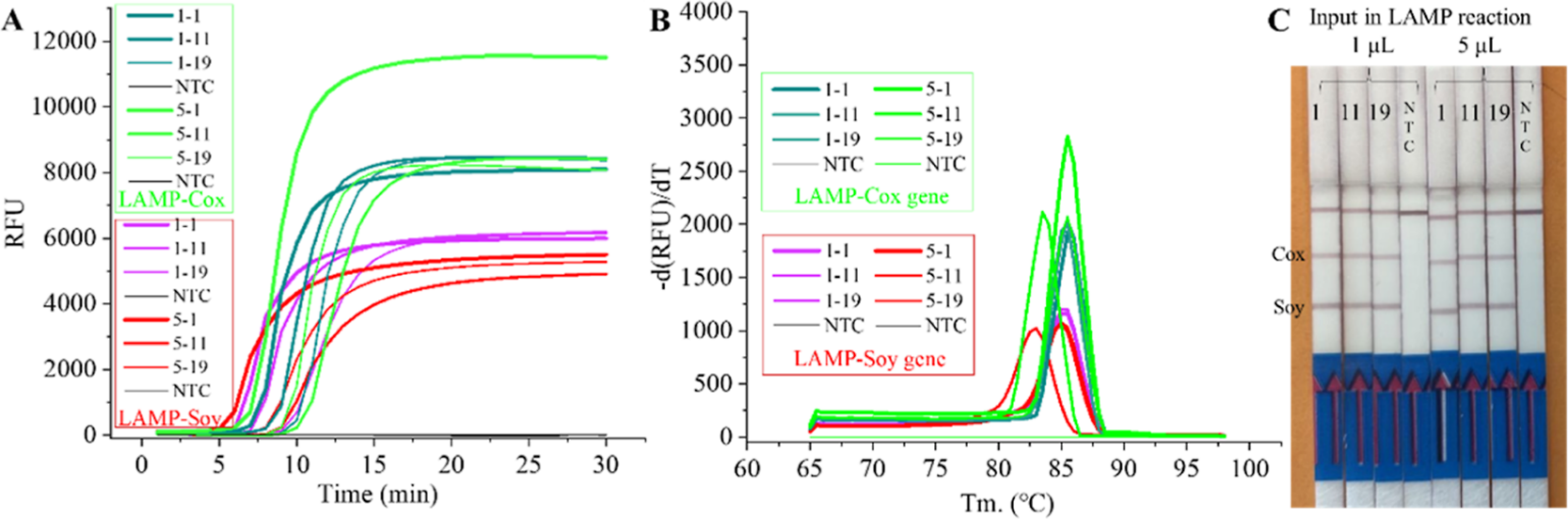
Food matrix effect in food sample #1 (100% soy), #11 (50% soy), #19 (>1% soy) with different inputs in LAMP reaction. (A) Amplification curves and (B) melting peaks as obtained in the Genie II. (C) Image after amplicons were diluted 200 times and analyzed with the LFD. Marks in the figures (A,B): 1–1 means 1 μL input of #1 sample and 5–1 means 5 μL input of #1 sample. NTC means no template control.

When developing methods to detect genetic material in complex matrices, food matrix effects influencing the results^17,19^ must be taken into account. If a detection method is not robust, it might lead to inaccurate test results. At the onset of this study, matrix effects were noted in the LAMP assay when testing the complex food matrices. As shown in Figure 3A and Supporting Information, Table S5, for the assessed commercial samples with high (#1), medium (#11), and low (#19) soybean content, the LAMP–Cox gene and LAMP–soy gene systems displayed positive amplification curves with both 1 and 5 μL input of the extracted DNA. The melting curves showed that the correct amplicons were produced as the melting temperature was the same as for pure DNA, namely, 85.5 ± 0.5 °C. However, when real samples were tested, specifically, a vegetarian diet 4 (sample #11 with 50% soy content), the melting peak of the amplicons obtained from 5 μL of extracted DNA was observed at 83.0 °C instead of 85.5 °C (Figure 3B). We expect this change to be a matrix effect. Indeed, when the template volume was decreased from 5 to 1 μL thereby also reducing the amount of inhibiting factors, the correct melting temperature of 85.5 °C was obtained again. Subsequent analysis on the LFD leads to robust and correct outcomes (Figure 3C).

**Figure 4:**
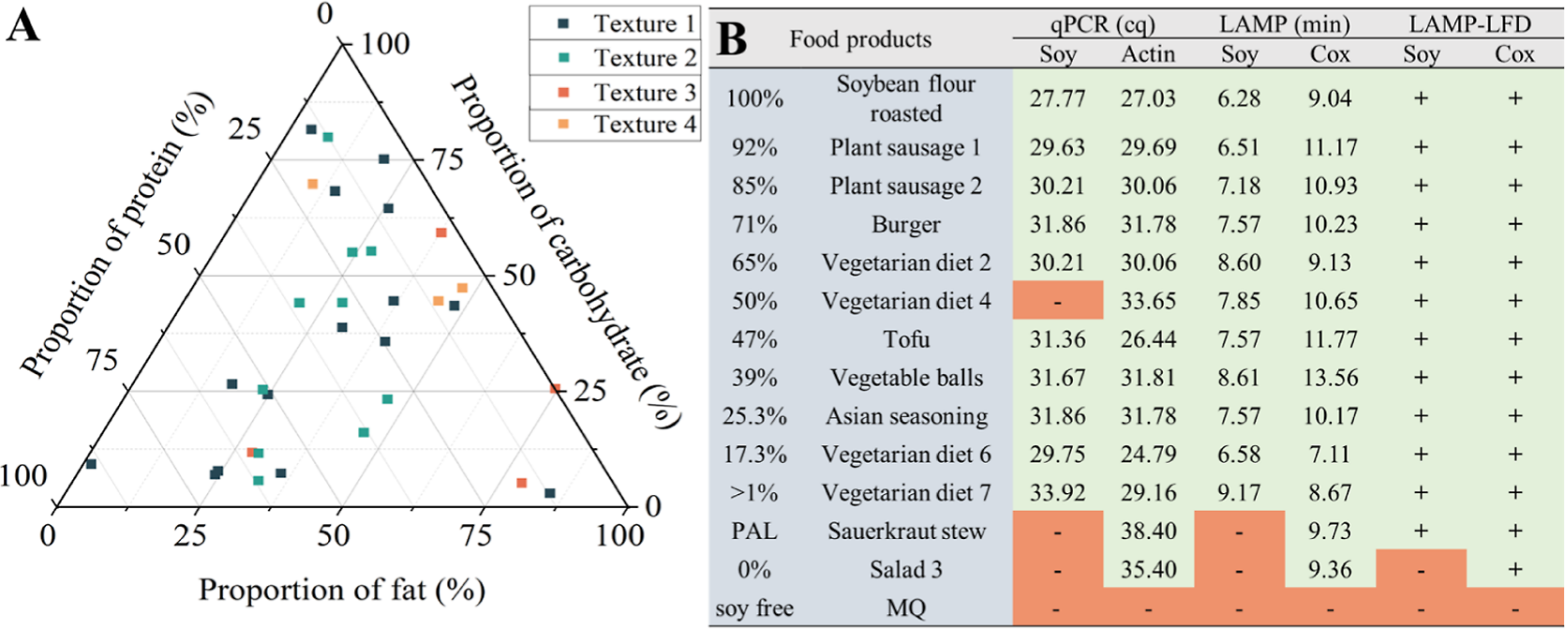
Study of food commodities. (A) The ternary plot analyzed food matrix distribution with texture 1 (hard solid, black squares), texture 2 (gelatinous and soft-solid, green squares), texture 3 (viscous liquid and emulsions, red squares), and texture 4 (liquid, orange squares). (B) The comparison of qPCR, LAMP, and LAMP–LFD assay by testing samples with different soy contents.

The results thus show that although there was an inhibitory effect in the LAMP assay when larger extract volume from a complex food product was used (Figure 4B), the LFD bands were not affected (Figure 4C). Overall, this indicates that the developed soy LAMP–LFD system with the Cox-gene control can be effectively applied to food products. In the following experiments, 2 μL of extract was chosen as the input for the LAMP and qPCR assays, after confirming this did not lead to a shift in the melting temperature and with efficient amplification when working with complex food extracts.

Thirty-two (32) commercial food products with different contents of soy were analyzed by LAMP and qPCR. Table S4 (Supporting Information) and Figure 4A display detailed information regarding the ingredient labeling, textures, and macronutrients of the samples. As shown in Tables 3 and S6, each sample was extracted twice and LAMP-analyzed in duplicate for high soy content samples, and with four replicates for low soy content samples. The results show that in the products that (according to the label) contain at least 1% soy (sample numbers 1–20), both the qPCR and LAMP correctly detect plant DNA, actin, and Cox, respectively. Soy was detected in 19 of these 20 samples by LAMP. For sample #17, after two rounds of DNA extraction, the results obtained by PCR and LAMP were inconsistent. The first extraction yielded positive results, while the second yielded negative results. This variability is attributed to the heterogeneous and complex nature of the salad matrix, which can lead to inconsistencies in DNA quality across different batches and operator interventions. The minimal soy DNA content in sample #17 further exacerbates these challenges. To substantiate these observations, additional replicate DNA extractions and a comparative evaluation of alternative DNA extraction techniques are required. Onlysample #18 (lasagna sauce) showed no amplification for soy with LAMP nor qPCR. The absence of amplification might be explained by the fact that lasagna sauce is a difficult matrix, regarding both constitution and processing, resulting in possible inhibition of the amplification assays and/or degradation of the target DNA. Another possibility is that the product was not labeled correctly.

Overall, the LAMP outcomes of these 20 samples were fully repeatable, except for sample #18. For one food product, #11, LAMP even outperformed qPCR as qPCR failed to amplify the soy gene, while using LAMP soy could be detected.

Regarding sample numbers 21–27, the PAL products, both qPCR and LAMP could correctly detect plant DNA. Both qPCR and LAMP did not detect soy in these samples, except in samples 21 and 25, where the results were inconclusive. To determine if a trace level amount is present, or whether it is false positive, amplification confirmation using alternative methods as immunoassays or LC–MS/MS is required.

In the soy-free plant-based samples #28 to #31, soy was not detected by qPCR nor by LAMP, except once in one sample of extraction 1 of coco milk (sample #31). Regarding the plant assays, the salad, #28, clearly showed to contain plant DNA, both by qPCR and LAMP; however this was less univocal for cream cheese (#29), cheese sauce (#30), and coco milk (#31). These ambiguous results for plant DNA using LAMP and qPCR in cream cheese (#29), cheese sauce (#30), and coco milk (#31) might be attributed to the fact that some foods underwent fermentation (cream cheese and cheese sauce) or ultrahigh temperature sterilization treatment to extend their shelf life (coco milk) during processing, possibly leading to degradation of the targeted DNA.

No soy was detected in the undeclared Vegan herbs sauce (32) by qPCR and LAMP, while only the LAMP assay was able to detect plant DNA in this sample. We speculated that the high fat content (78.6%) of this sample might have suppressed amplification of the actin gene using qPCR. The absence of amplification of the soy gene using both methods is most likely due to the fact that soy was not present. However, studies have also shown that high temperature, fat, and fermentation processes can reduce the yield and quality of plant DNA extraction and can produce DNA fragmentation, leading to negative results in qPCR analysis and LAMP.^18,19^

Next, the performance and the reliability of the soy and Cox gene targeting LAMP–LFD systems were checked by analysis of 13 real market food products (13 out of 32 in Table 4), with confirmation by qPCR. The selected products with varying soy content were very diverse, displaying different percentages of fat, carbohydrate, and protein (Figure 4A) that might have an effect on amplification efficiency. The detection results in Table 4 demonstrate our proposed LAMP could adequately distinguish soy in heterogeneous matrices, showing robustness for reliable on-site detection methods. As shown in Figure 4B, the results were consistent between the LAMP, LAMP–LFD (Figure S7), and qPCR, except for Sauerkraut stew. This sample was tested slightly positive by the LAMP–LFD, but this outcome was not confirmed by LAMP with fluorescent readout nor qPCR (Table 4).

**Table 4:**
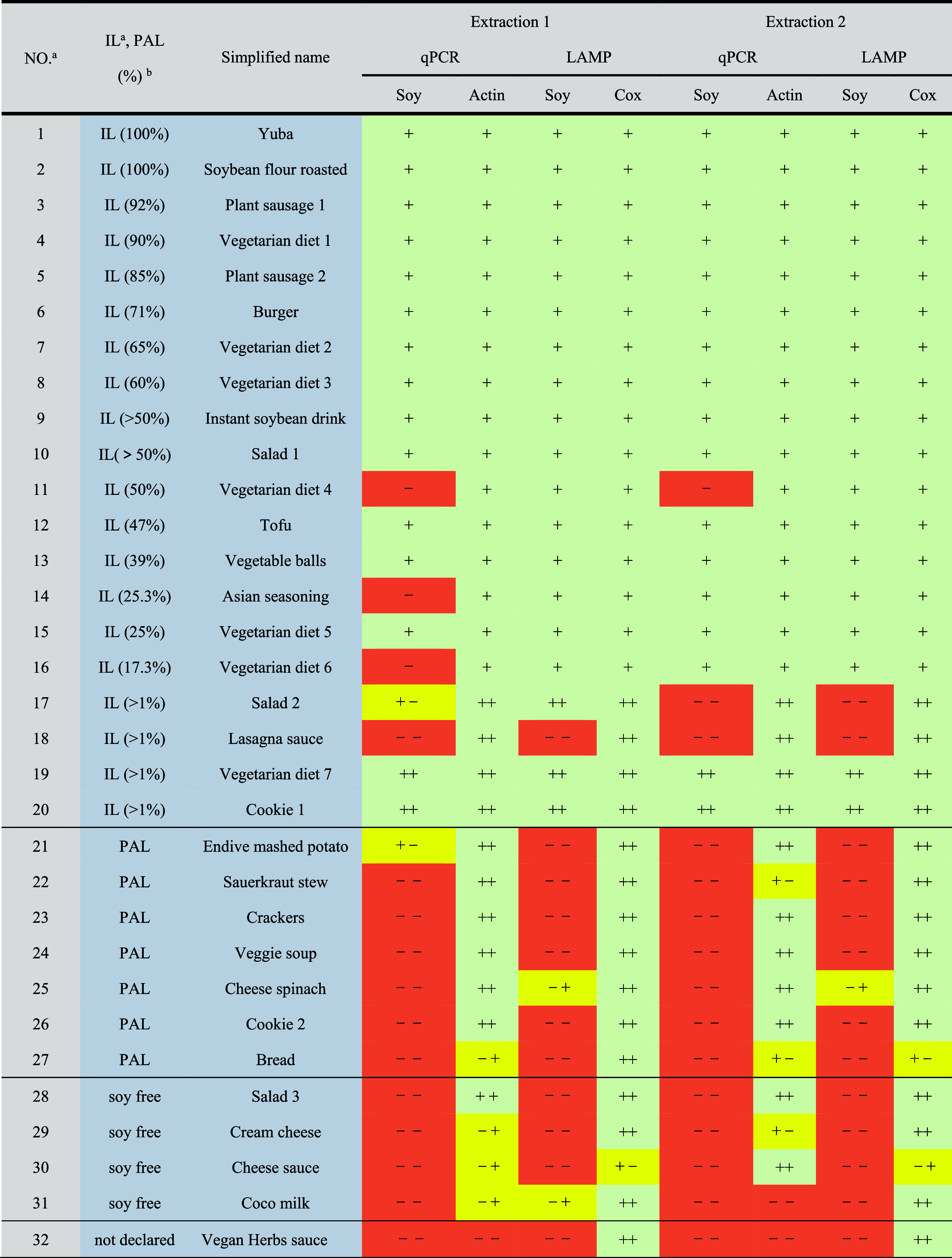
Summary of Real Market Samples Detected by LAMP and qPCR

a NOTE: same as the food information in Table S4, Supporting Information.

b Ingredient labeling, IL (content), precautionary allergen labeling, PAL. “+” represents that the sample was tested once, and it was positive. “–+” represents that the sample was tested twice with positive and negative results.

Notably, the entire LAMP–LFD detection procedure could be carried out within 20 min after obtaining DNA from the sample, which is highly efficient and applicable for on-site use. Besides, when comparing the performance of different assays for detection of soy based on DNA technology (Supporting Information, Table S7), we found that several investigations have documented a sensitive LOD, and some were based on laboratory approaches that are not conducive to on-site detection. Certain analytical techniques that reported on-site testing have omitted LOD determination. An important and critical aspect that is uniquely addressed in our study is that it has been comprehensively tested in a wide and representative collection of matrices, which demonstrates that it performs well in terms of simplicity, sensitivity, and applicability as well as unveils its limitations.

### 3.5 On-Site Applicable Readout of the Newly Developed LAMP–LFD

**Figure 5:**
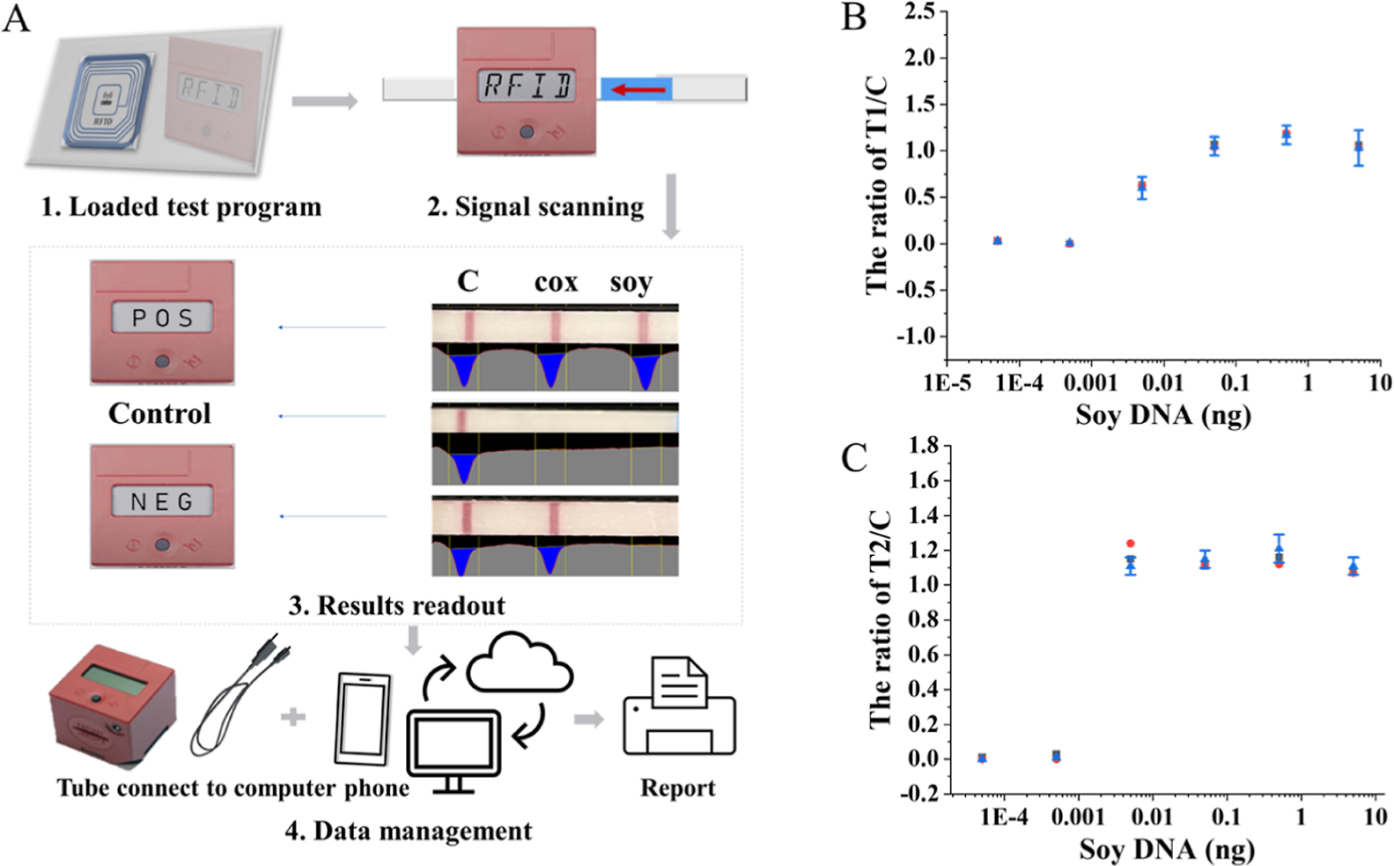
Workflow of the LAMP–LFD assay equipped with the digital Cube Reader and RFID-tag (A); the ratio of color intensity T1/C (B) and T2/C (C), where T1 is Cox and T2 is soy. Error bars represent the RSD by calculating the color intensity of the LAMP–LFD system three different times using the digital Cube Reader.

LFD offers users both simple visual interpretation and semiquantitative analysis when using optical readers to analyze the color intensities of the T-line and C-line.^11^ A risk of visual inspection, or even semiquantification with a smartphone (when not using a dedicated adaptor) is misinterpretation due to differences in color perception or variable ambient light.^20^ Therefore, to report results, analyze data, and share allergen information more accurately, a dedicated optical reader was used in the present study, i.e., a Cube Reader. As shown in Figure 5A, the LOD of the LAMP assay was set as the detection threshold and then the test program was loaded in the digital cube. Next, when the LFD was scanned by the Cube to measure the color intensities of the T-lines (soy and Cox) and the C-line, the color intensities were automatically calculated against the detection threshold. For example, if a plant sample with a soy content exceeds the threshold, the result will be reported as positive (T-lines and C-line are clearly red); if a soy-free plant sample is tested, the result will be reported as negative (T1-line (Cox) is red and T2-line (soy) is colorless and C-line is red); in a sample without plant genes, there should only be one observable red line, i.e., that of the C-line. Then, to validate the expected application of semiquantitative detection, different amounts of pure soybean DNA were tested by duplex LAMP–LFD using digital Cube analysis, and it proved reliable comparing with laboratory-based detection (relative standard deviation, RSD < 3.9%) (Figure 5B,C and Table S8). Finally, the small and cost-effective reader can easily be connected to a phone or laptop, store and share analytical data with consumers, alerting consumers of the potential occurrence of traces of allergenic ingredients.

The incidence of food allergies has significantly increased worldwide, with soy allergies presenting a significant public health and food safety issue, potentially leading to life-threatening situations. Due to the varying legislation on allergen limits in several countries, and even the varying tolerance of individuals with allergies to ingested food, it is difficult to directly determine whether food is safe. Developments of highly sensitive, specific, and practical detection methods for soy allergen remain imperative for food producers, restaurants, and inspectors, or even consumers. Driven by this fact, this study reports on the development of a robust duplex LAMP–LFD assay for the rapid detection of soy-derived genes in complex food matrices.

The LAMP assays targeting soy and Cox genes were successfully evaluated by testing both pure soy DNA and spiked samples with high sensitivity and specificity. Although DNA detection does not directly identify allergenic proteins, it fulfills regulatory requirements for ingredient declaration and provides critical risk alerts for allergen management. DNA-based detection complements protein-based methods by providing a rapid (≤20 min), sensitive (LOD 5 × 10^–4^ ng) and matrix-tolerant screening tool for unintended soy introduction, crucial for preventing cross-contamination in complex food supply chains. In addition, without requiring any sophisticated instrument, the duplex LFD could always interpret the LAMP products accurately within approximately 20 min, reporting comparable results as LAMP with fluorescent detection. Moreover, this assay employed the Cox gene as a reference gene to confirm correct DNA extraction and amplification, to avoid false negative results and confirm correct performance. By changing target genes and the corresponding LAMP primers, the developed strategy has the potential to advance ready-to-use smart devices for detecting other food allergens.

Although the proposed LAMP–LFD assay has been proven to have potential, several practical limitations must be addressed to achieve strong on-site applicability. For example, here laboratory-based DNA extraction methods were applied that are incompatible with on-site applications. Therefore, more simplified DNA extraction methods will be required. Besides, this strategy has a risk of aerosol contamination when the LAMP amplification products are transferred to LFD. A closed system therefore is required. The above might be mediated by using microfluidic chips and three-dimensional printing technology to further develop an integrated and user-friendly LAMP–LFD platform with the assistance of digital cubes for detection, interpretation, and data storage.^21^

## Supporting Information Available

Schematic of LAMP products detected by duplex LFD; optimization of the LAMP assays; specificity of the LAMP assay; sensitivity for the detection of pure soy DNA; optimization of LAMP products for LFD detection; LOD determination of the LAMP–LFD system; results of LAMP–LFD for the detection of commercial food samples with different soy contents; sequences of the primers for qPCR assay; sequences of the primers for the LAMP assay; eighteen plant species’ information and LAMP detection of their extracted DNA in a specificity test; complex food samples used in this assay; amplification data of food matrix effect in food samples with different input in LAMP reaction; amplification data of real market samples detected by LAMP and qPCR; comparison of different detection assays of soy allergen based on DNA technology; and semiquantitative results tested by LAMP–LFD assay equipped with a digital cube and RFID-tag (PDF)
