# Supplementary material for: Reference Gene-Assisted LAMP–LFD for Sensitive and Specific Detection of Soy DNA as a Marker for Allergen Presence in Complex Food Products
Source: J Agric Food Chem. 2025 Apr 24;73(18):11340–50. doi: 10.1021/acs.jafc.5c01463 (PMC12063182; doi:10.1021/acs.jafc.5c01463)
Supplement: Supplementary file 1 — jf5c01463_si_001.pdf [file jf5c01463_si_001.pdf]

Supporting Information

Reference gene-assisted LAMP-LFD for sensitive and specific detection of soy DNA as a marker for allergen presence in complex food products

Qiaofeng Li [a, b, c, d, †], Marleen M. Voorhuijzen-Harink [b, †], Dianpeng Han [c], Bas J. Fronen [b], Richard van Hoof [b], Ming Chen [a], Zhouping Wang [d], Toine F. H. Bovee [b], Zhixian Gao [c, *], Gert IJ. Salentijn [b, e *]

[a] Department of Clinical Laboratory Medicine, Southwest Hospital, Third Military Medical University (Army Medical University), 30 Gaotanyan, Shapingba District, Chongqing 400038, China

[b] Wageningen Food Safety Research, Wageningen University & Research, P.O. Box 230, 6700, AE, Wageningen, the Netherlands

[c] Tianjin Key Laboratory of Risk Assessment and Control Technology for Environment and Food Safety, Military Medical Sciences Academy, Tianjin 300050, China

[d] State Key Laboratory of Food Science and Technology, Jiangnan University, Wuxi 214122, China

[e] Laboratory of Organic Chemistry, Wageningen University, Wageningen, 6708, WE, the Netherlands

† These authors contributed equally to this work.

* Corresponding author: Gert IJ. Salentijn, Zhixian Gao

Address: Wageningen Food Safety Research, Wageningen University & Research, P.O. Box 230, 6700 AE Wageningen, the Netherlands; Tianjin Key Laboratory of Risk Assessment and Control Technology for Environment and Food Safety, Military Medical Sciences Academy, Tianjin 300050, China.

Email: gert.salentijn@wur.nl (Dr. Gert IJ. Salentijn); gaozhx@163.com (Dr. Zhixian Gao)

Content

Part 1 Figures 3

Figure S1. Schematic of LAMP products detected by duplex LFD. 3

Figure S2. Optimization of the LAMP assays. 4

Figure S3. The specificity of the LAMP assay. 5

Figure S4. Sensitivity for the detection of pure soy DNA. 6

Figure S5. The optimization of LAMP products for LFD detection. 7

Figure S6. LOD determination of the LAMP-LFD system. 8

Figure S7. Results of LAMP-LFD for the detection of commercial food samples with different soy content. 9

Part 2 Tables 10

Table S1. Sequences of the primers for qPCR assay. 10

Table S2. Sequences of the primers for the LAMP assay 11

Table S3. 18 plant species information and LAMP detection of their extracted DNA in specificity test. 12

Table S4. The complex food samples used in this assay. 13

Table S5. Amplification data of food matrix effect in food sample with different input in LAMP reaction. 14

Table S6. Amplification data of real market samples detected by LAMP and qPCR. 15

Table S7. Comparison of different detection assays of soy allergen based on DNA technology. 16

Table S8. The semi quantitative results tested by LAMP-LFD assay equipped with digital cube and RFID-tag 18

References: 19

Part 1 Figures

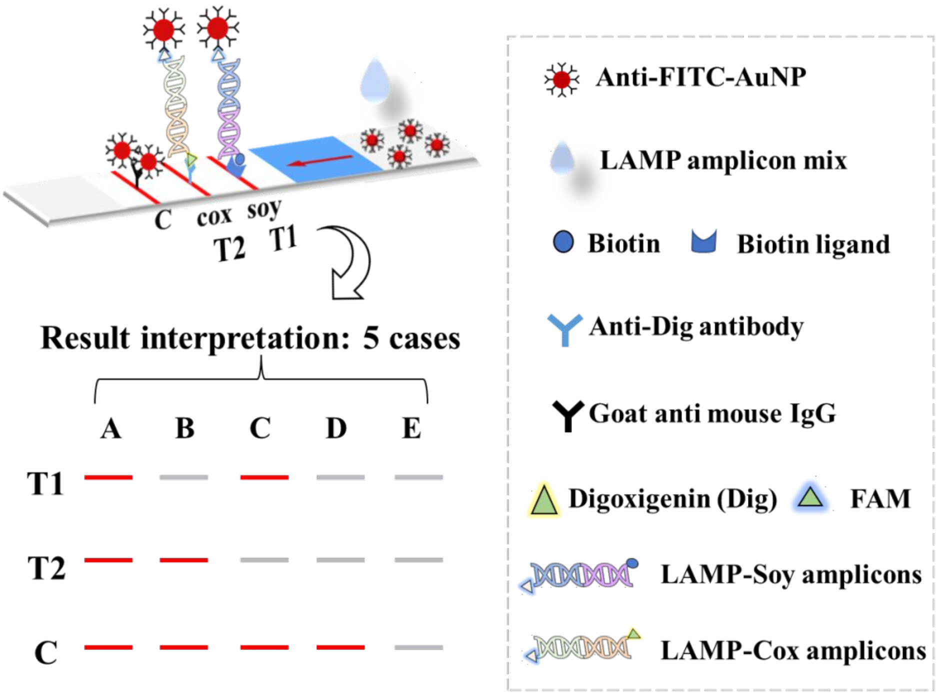

Figure S1. Schematic of LAMP products detected by duplex LFD.

100 μL of the amplification products were adsorbed to the sample pad of the LFD strip and migrated by capillary action. Subsequently, FAM- and biotin-labeled LAMP-soy amplicons, captured by gold particles via an anti-FITC gold-labeled antibody, are anchored on test line 1 (T1) by the biotin ligand. FAM- and Dig-labeled LAMP-Cox amplicons, captured by gold particles via an anti-FITC gold-labeled antibody, are captured on test line 2 (T2) by the anti-Dig antibody. Free gold-labeled antibodies are anchored at the control line (C line).

The results were interpreted into 5 cases: if the T1, T2 and C lines all show red color, it means there is soy present in the analyte (case A). If the T2 and C lines are visible, it means no soy is detected above the detection limit, and that the test was run correctly (case B). If only T1 and C lines show red color, the result must be considered invalid, as no plant material was detected, yet a positive hit was found on soy (case C). If only a C line is visible, it indicates that no plant-derived gene was detected in the analyte, or poor DNA extraction/amplification when plant material is in fact present – which can be valid if indeed no plant material was present (case D). An invisible C line after the incubation period means an invalid result, as the LFD was not developed properly (case E).

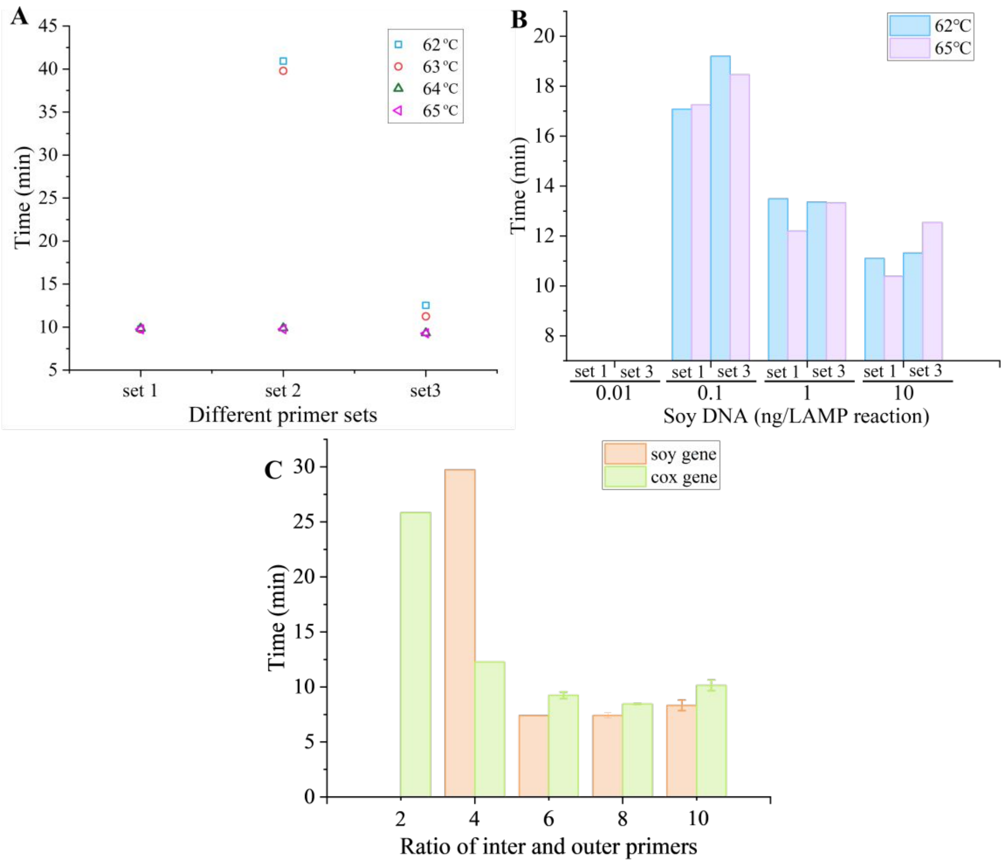

Figure S2. Optimization of the LAMP assays.

(A) Soy primer sets; (B) LAMP temperature; (C) Ratio of inter and outer primers. No amplification (time) reported means the LAMP system did not work when 0.01 ng Soy DNA was input in this experiment condition (in Fig. S2B) and when the ratio of inner and outer primers was 2 (in Fig. S2C).

Fig. S2A: three soy primer sets were tested at different temperatures by LAMP protocol with 10 ng soy DNA as the template and each temperature (62℃-65℃) was tested once per set. All three primer sets could be used to successfully amplify the target genes, but the large relative standard deviation generated by set 2 by small temperature changes points towards a less robust assay. Therefore, set 2 was excluded at this stage.

Fig. S2B: set 1 and set 3 were compared by testing different input amounts of soy DNA at 62℃ and 65℃ and the results were similar based on this preliminary optimization. Shorter targets enhance compatibility with degraded templates, which offers greater advantages in processed food products [1]. Therefore, due to the shorter amplicon size, set 1 was used in the subsequent experiments.

Fig. S2C: based on the optimized conditions, the inner and outer primer ratios of LAMP-soy and LAMP-Cox systems were optimized with 10 ng/uL soybean as a template, each set was tested in triplicate. The results show that the amplification time was shortest for the LAMP-soy and LAMP-Cox systems at the ratio of 6 and 8, respectively. Therefore, we chose these two optimal parameters for subsequent experiments.

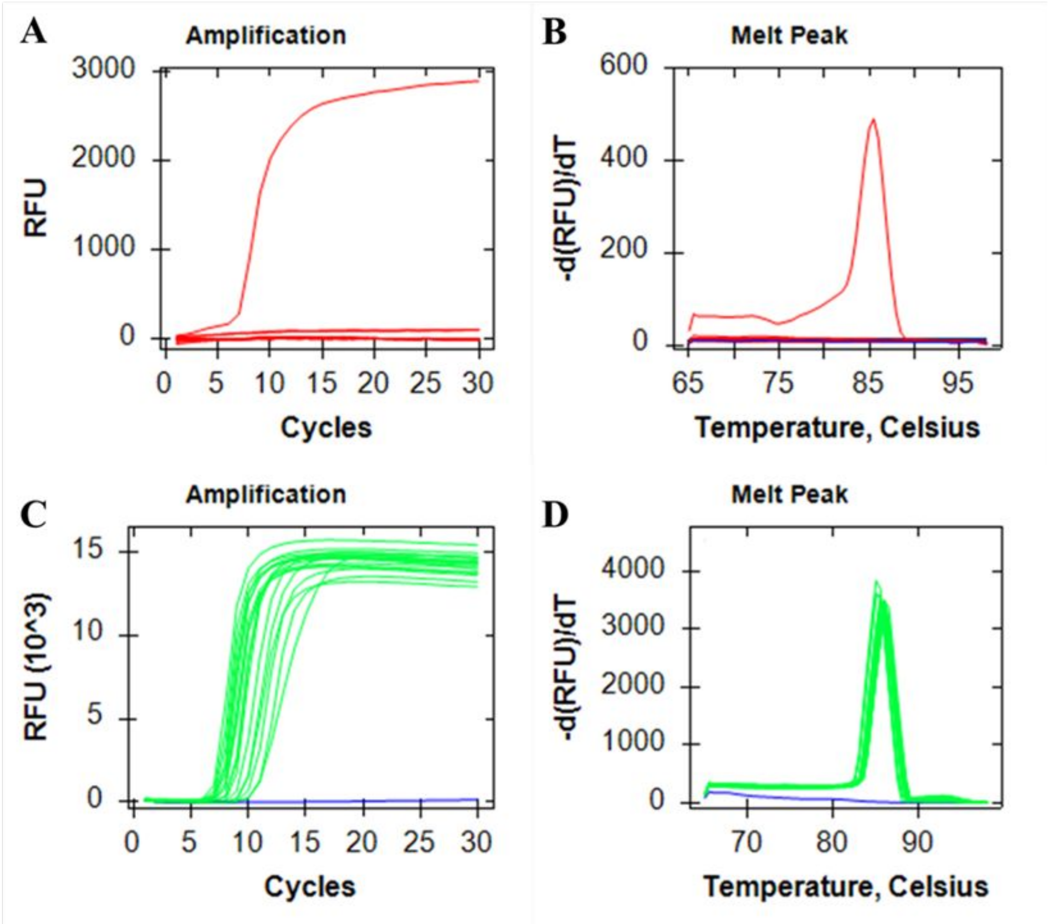

Figure S3. The specificity of the LAMP assay.

(A) The amplification and (B) melt curve of LAMP-soy gene system (red lines); (C) The amplification and (D) melt curve of LAMP-Cox gene system (green lines). RFU: Relative fluorescence units.

The extracted DNA from 18 plant species commonly used in food products were examined by LAMP assay (Table 1). Only 1 positive result (soy DNA) was obtained when all the templates were tested by LAMP-soy gene system, while all 18 plant species showed positive results when implementing LAMP-Cox gene system. As no template control (NTC), the nuclease-free water was always tested negative in LAMP assay, which was marked in blue color in the Figure.

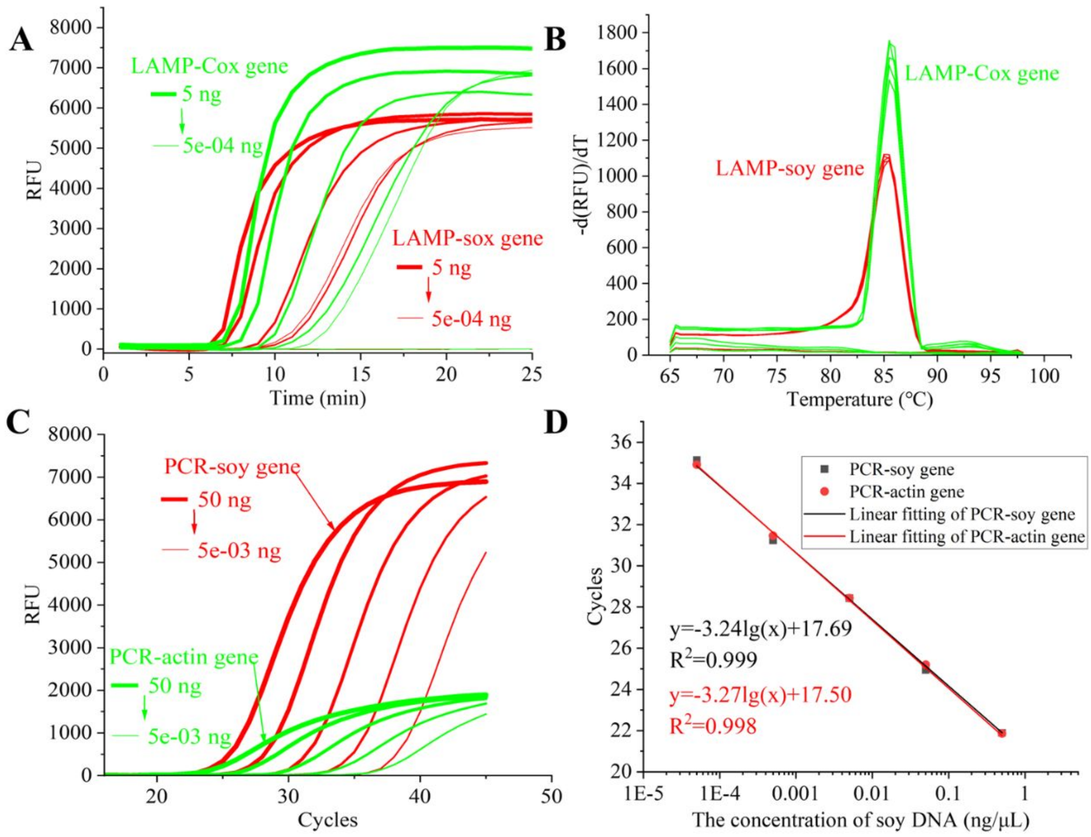

Figure S4. Sensitivity for the detection of pure soy DNA.

(A) The amplification curves and (B) melt peaks of LAMP assay; (C) The amplification curves and (D) calibration curve of qPCR assay.

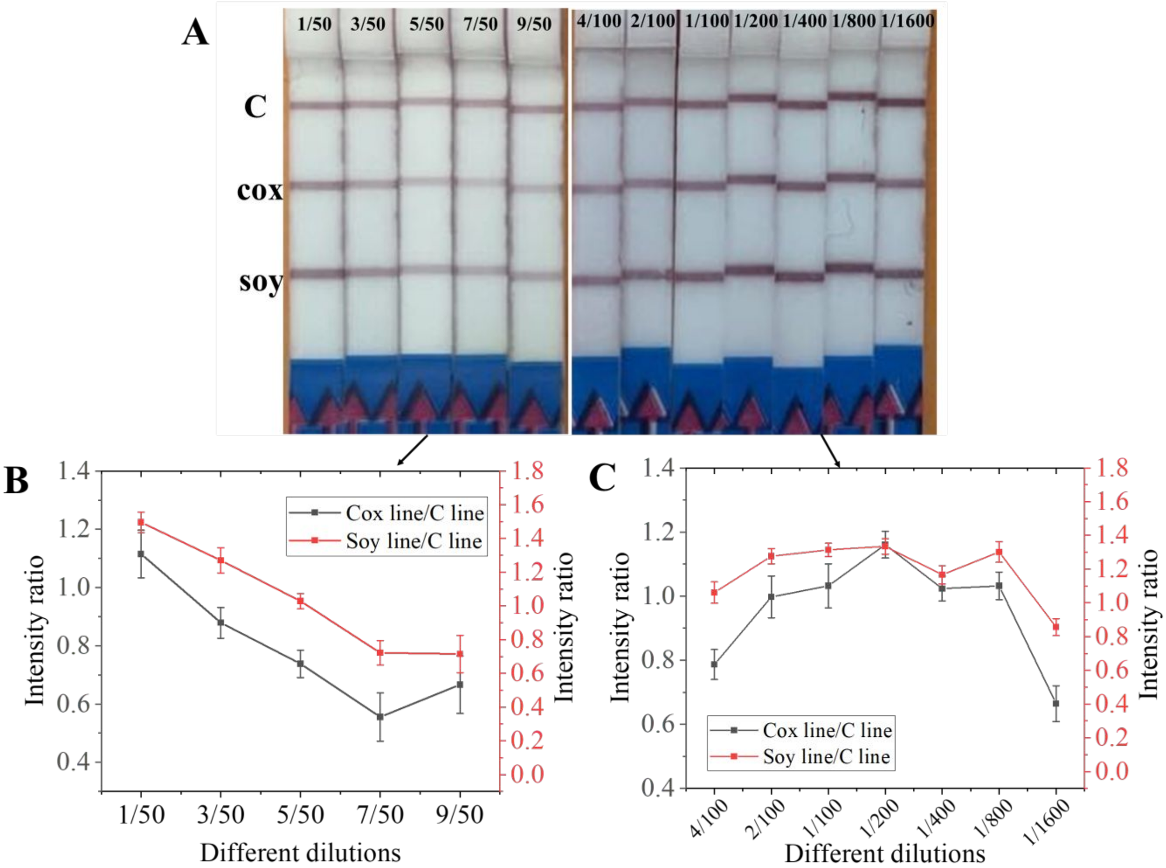

Figure S5. The optimization of LAMP products for LFD detection.

(A) The images after amplicons were diluted and analyzed with the LFD; (B) and (C) Quantitative analysis of the grayscale values of each test strip using ImageJ software. Error bars represent the RSD by calculating the color intensity from the strips three different times using ImageJ software.

In Figure S5A, 1/50, 3/50, 5/50, 7/50, 9/50 represents that 1 μL, 3 μL, 5 μL, 7 μL, 9 μL of LAMP-Soy amplicons/LAMP-Cox amplicons were added, then the running buffer was added to reach a final volume of 50 μL running solution (For example, 1/50 means 50 μL running solution including 1 μL of LAMP-Soy amplicons, 1 μL of LAMP-Cox amplicons plus 48 μL of running buffer), and developed on the LFD subsequently. However, results show that the color intensities of the test lines decreased with the increase of amplicons, indicating the high-dose hook effect of sandwich lateral flow immunoassays[2]. Therefore, we increased the running solution to 100 μL and diluted the amplicons with buffer to the dilutions of 4/100, 2/100, 1/100, 1/200, 1/400, 1/800, 1/1600, respectively. Finally, the 1/200 dilution was chosen for the subsequent experiment.

In order to make the high-dose hook effect more clear, we have quantitatively analyzed the grayscale values of each test strip using ImageJ software, and created a new graph (grey scale intensity as a function of dilution factor) to visually demonstrate the hook effect, and how dilution can mitigate it. As shown in Figure S5B and C, the x-axis represents different dilutions as shown in Figure S5A, and the y-axis represents the ratio of grayscale values on the T-line and C-line (for example, the Cox line/C line represents the intensity ratio of grayscale from Cox line and C line). As expected, the results were consistent with the visual qualitative analysis of the test strips.

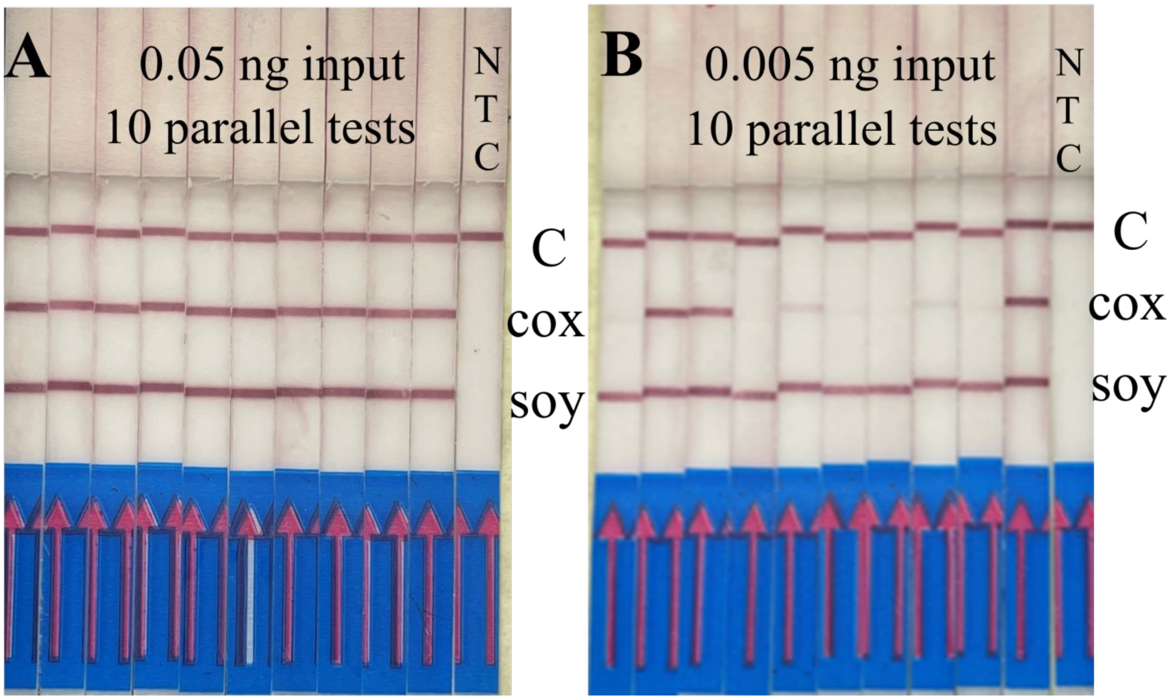

Figure S6. LOD determination of the LAMP-LFD system.

(A) 0.05 ng and (B) 0.005 ng input per LAMP reaction.

NTC means no template control. In the LFD running, a 200-fold dilution of the LAMP reaction product was analyzed, and the test was carried out by 10 parallel tests. The result showed that the LOD of LAMP-LFD assay was 5×10^{-2} ng per reaction since there were only 3 out of 10 the Cox T-lines showed obviously positive in LAMP-LFD system with 0.005 ng input per LAMP reaction.

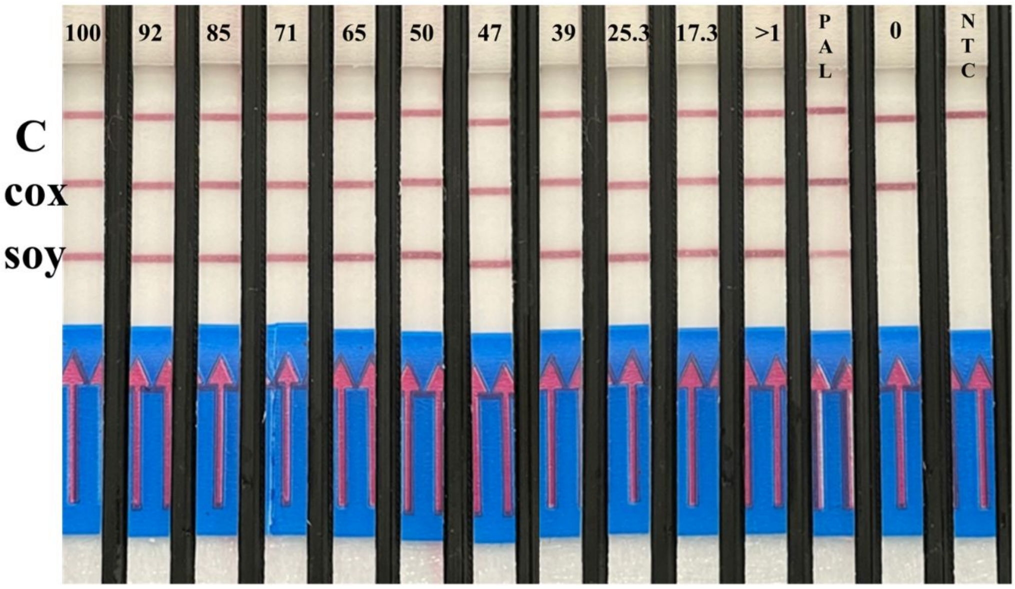

Soy content		100%	92%	85%	71%	65%	50%	47%	39%	25.3%	17.3%	>1%	PAL	0%	NTC
Amplificatio	Cox	9.00	10.15	10.30	10.51	11.23	12.45	11.21	12.55	12.24	13.00	12.34	10.45	11.15	-
n time	Soy	7.00	7.45	8.00	7.54	11.10	10.15	8.43	9.50	9.03	10.14	9.48	13.10	-	-
Cube	Cox	+	+	+	+	+	+	+	+	+	+	+	+	+	-
readout	Soy	+	+	+	+	+	+	+	+	+	+	+	+	-	-

Figure S7. Results of LAMP-LFD for the detection of commercial food samples with different soy content.

(On top of the image from left to right: 100%, 92%, 85%, 71%, 65%, 50%, 47%, 39%, 25.3%, 17.3%, 1%, PAL, 0%, NTC. On bottom of the image are the amplification times and digital cube reader results for detection of soy and Cox gene, + positive, - negative)

Part 2 Tables

Table S1. Sequences of the primers for qPCR assay.

Target	Name	Sequence 5’-3’
Plant gene [3]	Act-F	CAAGCAGCATGAAGATCAAGGT
	Act-R	CACATCTGTTGGAAAGTGCTGAG
	Act-probe	FAM-CCTCCAATCCAGACACTGTACTTYCTCTC-TAMRA
Soy-specific gene [4]	Lec-F	CCAGCTTCGCCGCTTCCTTC
	Lec-R	GAAGGCAAGCCCATCTGCAAGCC
	Lec-probe	FAM-CTTCACCTTCTATGCCCCTGACAC-TAMRA

Table S2. Sequences of the primers for the LAMP assay

Primer name	Sequence (5'→3')
Soy Primer set 1	Sequence (5'→3')
F3	TCATAATCTAATTTTCGGGCTAA
B3	AAAGAGAGTGACGATGTCAT
FIP	AGCCAATTCGAAGTTCCAAGATAAAGTCTTCTATATCTTGGGATACAACA
BIP	GGACGGGGTCAGTATTACGCGAAGATTCCTAATAATACCCAATGA
LoopF	GCTATTCTGATAGATAGTGGCAAAC
LoopB	TTATGGTTAACCCGGCCCCTT
biotin-FIP	biotin-AGCCAATTCGAAGTTCCAAGATAAAGTCTTCTATATCTTGGGATACAACA
FAM-LoopF	FAM-GCTATTCTGATAGATAGTGGCAAAC
Soy Primer set 2	Sequence (5'→3')
F3	CTAATTTTCGGGCTAATAGTCTT
B3	AAAGAGAGTGACGATGTCAT
FTP	AGCCAATTCGAAGTTCCAAGATAAATTGGGATACAACAATCTAATGTTTG
BIP	GGACGGGGTCAGTATTACGCGAAGATTCCTAATAATACCCAATGA
LoopB	CCCGGCCCCTTCTGATT
Soy Primer set 3 [5]	Sequence (5'→3')
F3	CCGAGTCTGCTGCCGTAT
B3	ATGAGATTGAGTTCCACGCA
FTP	GGGGTCAGTATTACGCCTCTGACAAAGAAAGAGAGTGACGATG
BIP	TCTGATAGATAGTGGCAAACATTAGTTGCTGCTATTCCATCTATTCAT
LoopF	TTCTGATTCCGCTCATTGG
LoopB	CAAGATATAGAAGACTATTAGCCCG
Cox Primer set [6]	Sequence (5'→3')
F3	TATGGGAGCCGTTTTTGC
B3	AACTGCTAAGRGCATTCC
FIP	ATGGATTTGRCCTAAAGTTTCAGGGCAGGATTTCACTATTGGGT
BIP	TGCATTTCTTAGGGCTTTCGGATCCRGCGTAAGCATCTG
LoopF	ATGTCCGACCAAAGATTTTACC
LoopB	GTATGCCACGTCGCATTCC
Dig-FIP	DIG-ATGGATTTGRCCTAAAGTTTCAGGGCAGGATTTCACTATTGGGT
FAM-LoopF	FAM-ATGTCCGACCAAAGATTTTACC

Table S3. 18 plant species information and LAMP detection of their extracted DNA in specificity test.

No	Species Name	Family	Common Name	C [a] ng/μL	LAMP			
					Soy		Cox	
					Time [b]	Tm [c]	Time	Tm
1	*Sinapsis alba*	*Brassicaceae*	White mustard	10	--	--	9.84	85.0
2	*Pistacia vera*	*Anacardiaceae*	Pistachio	10	--	--	7.31	85.5
3	*Sesamum indicum*	*Pedaliaceae*	Sesame	6	--	--	7.06	85.0
4	*Apium graveolens, var secalinum*	*Apiaceae*	Celeriac	10	--	--	8.41	86.0
5	*Triticum spelta, Sammy*	*Poaceae*	Spelt	10	--	--	9.21	85.5
6	*Triticum aestivum*	*Poaceae*	Common wheat	10	--	--	9.8	86.0
7	*Portulaca oleracea L.*	*Portulacaceae*	Common purslane	10	--	--	11.03	85.5
8	*Carya illinoinensis*	*Juglandaceae*	Pecan	10	--	--	7.57	86.0
9	*Bertholletia excelsa*	*Lecythidaceae*	Brazil nut	10	--	--	7.51	85.5
10	*Macadamia sp.*	*Proteaceae*	Macadamia	10	--	--	7.75	86.0
11	*Juglans regia*	*Juglandaceae*	Walnut	10	--	--	8.1	86.0
12	*Corylus avellana*	*Betulaceae*	Hazelnut	10	--	--	8.13	86.0
13	*Apium graveolens Dulce Group*	*Apiaceae*	Celery	10	--	--	8.03	86.0
14	*Lupinus albus*	*Fabaceae*	White Lupine	10	--	--	7.18	86.0
15	*Arachis hypogaea*	*Fabaceae*	Peanut	10	--	--	8.46	86.0
16	*Phaseolus vulgaris L.*	*Fabaceae*	Kindey bean	10	--	--	8.73	86.0
17	*Pisum sativum*	*Fabaceae*	Pea	10	--	--	10.25	85.5
18	*Glycine max*	*Fabaceae*	Soybean	10	7.08	85.5	10.13	86.0

NOTE: [a] Concentration of the plant DNA; [b] Amplification time; [c] Melt temperature.

Table S4. The complex food samples used in this assay.

No.	Food matrix groups	Fat (%)[a]	Carbohydrate (%)[a]	Protein (%)[a]	Texture[b]	IL/PAL[c]
1	Yuba	24.8	24.3	50.9	1	IL (100%)
2	soybean flour roasted	23.3	25.6	51.2	2	IL (100%)
3	Plant sausage 1	45.6	16.3	38.1	2	IL (92%)
4	Vegetarian diet 1	24.3	7.8	67.8	1	IL (90%)
5	Plant sausage 1	24.2	7.0	68.7	1	IL (85%)
6	Burger	46.2	23.5	30.4	2	IL (71%)
7	Vegetarian diet 2	32.3	5.9	61.7	2	IL (65%)
8	Vegetarian diet 3	17.4	26.6	56.0	1	IL (60%)
9	instant soybean drink	9.8	69.7	20.5	4	IL (>50%)
10	Salad 1	35.6	7.3	57.1	1	IL(>50%)
11	Vegetarian diet 4	20.3	44.4	35.4	2	IL (50%)
12	Tofu	29.4	11.8	58.8	2	IL (47%)
13	Vegetable balls	28.0	12.0	60.0	3	IL (39%)
14	Asian seasoning	39.6	35.8	24.6	1	IL (25.3%)
15	Vegetarian diet 5	1.4	9.3	89.4	1	IL (25%)
16	Vegetarian diet 6	36.7	44.6	18.7	1	IL (17.3%)
17	Salad 2	47.9	43.6	8.6	1	IL (>1%)
18	Lasagna sauce	7.4	80.2	12.5	2	IL (>1%)
19	Vegetarian diet 7	30.6	38.9	30.6	1	IL (>1%)
20	Cookie 1	19.7	75.2	5.1	1	IL (>1%)
21	Endive mashed potato	24.1	55.3	20.6	2	PAL
22	Sauerkraut stew	27.3	55.6	17.2	2	PAL
23	Crackers	14.6	68.3	17.1	1	PAL
24	Veggie soup	44.4	44.4	11.1	4	PAL
25	Cheese spinach	27.8	44.4	27.8	2	PAL
26	Cookie 2	25.8	64.6	9.6	1	PAL
27	Bread	3.8	81.6	14.6	1	PAL
28	Salad 3	84.8	3.0	12.1	1	soy free
29	Cream cheese	74.2	25.8	0.0	3	soy free
30	Cheese sauce	37.4	59.5	3.1	3	soy free
31	Coco milk	47.3	47.3	5.5	4	soy free
32	Vegan Herbs sauce	78.6	5.4	16.1	3	not declared

Note: [a] Calculated based on the reference value of every 100g of nutrients on the food label. [b] Based on scale 1–4 with 1 hard solid, 2 gelatinous and soft-solid, 3 viscous liquid and emulsions, 4. Liquid. [c] May contain soy. Ingredient labeling, IL (content)/Precautionary allergen labeling, PAL[c].

The selection of such a diverse range of real food products and the ternary plot analyzing food matrix distribution with different textures was to verify the broad applicability of the established method, and also to identify potential factors that may affect the detection results. The results of the food commodities can be found in Table 3 and Figure 4 in the main text.

Table S5. Amplification data of food matrix effect in food sample with different input in LAMP reaction.

DNA input	1 μL				5 μL			
Sample	#1	#11	#19	NTC	#1	#11	#19	NTC
Time (min)	6.34	7.53	9.93	N/A	5.84	9.52	8.52	N/A
Tm. (℃)	85.5	85.5	85.5	None	85	83	85	None
Time (min)	7.33	8.68	10.12	N/A	6.9	10.52	9.33	N/A
Tm. (℃)	85.5	85.5	85.5	None	85.5	83.5	85.5	None

Note: "Time" means amplification time (The unit is minute). The "Tm." represents the melting temperature (The unit is degrees C). The "N/A" represents no detectable template. The "None" represents no melting temperature was reported. The "NTC" represents no template control.

Table S6. Amplification data of real market samples detected by LAMP and qPCR.

NO	IL [a], PAL (%) [b]	Simplified name	qPCR Soy	qPCR Actin	LAMP Soy		LAMP Cox	
			Cq.	Cq.	Time [c]	Tm. [d]	Time	Tm.
1	IL (100%)	Yuba	25.66 (2/2) [e]	25.22 (2/2)	5.85 (2/2) [f]	85 (2/2) [g]	9.00 (2/2)	86 (2/2)
2	IL (100%)	Soybean flour roasted	27.77 (2/2)	26.99 (2/2)	6.24 (2/2)	85 (2/2)	8.97 (2/2)	86 (2/2)
3	IL (92%)	Plant sausage 1	29.63 (2/2)	29.69 (2/2)	6.51 (2/2)	85 (2/2)	11.17 (2/2)	86 (2/2)
4	IL (90%)	Vegetarian diet 1	32.52 (2/2)	33.56 (2/2)	9.77 (2/2)	85 (2/2)	12.29 (2/2)	86 (2/2)
5	IL (85%)	Plant sausage 2	30.21 (2/2)	30.06 (2/2)	7.18 (2/2)	85 (2/2)	10.93 (2/2)	86 (2/2)
6	IL (71%)	Burger	31.86 (2/2)	31.78 (2/2)	7.57 (2/2)	85.5 (2/2)	10.23 (2/2)	86 (2/2)
7	IL (65%)	Vegetarian diet 2	30.21 (2/2)	30.06 (2/2)	8.60 (2/2)	85.5 (2/2)	9.13 (2/2)	86 (2/2)
8	IL (60%)	Vegetarian diet 3	31.59 (2/2)	32.96 (2/2)	5.47 (2/2)	85 (2/2)	11.74 (2/2)	86 (2/2)
9	IL (>50%)	Instant soybean drink	31.45 (2/2)	34.48 (2/2)	6.03 (2/2)	85 (2/2)	9.36 (2/2)	86 (2/2)
10	IL(> 50%)	Salad 1	27.18 (2/2)	26.78 (2/2)	8.21 (2/2)	85 (2/2)	12.77 (2/2)	86 (2/2)
11	IL (50%)	Vegetarian diet 4	–	33.65 (2/2)	7.85 (2/2)	85 (2/2)	10.65 (2/2)	85.5 (2/2)
12	IL (47%)	Tofu	31.36 (2/2)	26.44 (2/2)	7.57 (2/2)	85 (2/2)	11.77 (2/2)	86 (2/2)
13	IL (39%)	Vegetable balls	31.67 (2/2)	31.81 (2/2)	8.61 (2/2)	85 (2/2)	13.56 (2/2)	86 (2/2)
14	IL (25.3%)	Asian seasoning	31.86 (1/2)	31.78 (2/2)	7.57 (2/2)	85 (2/2)	10.17 (2/2)	86 (2/2)
15	IL (25%)	Vegetarian diet 5	36.9 (2/2)	34.21 (2/2)	7.05 (2/2)	85 (2/2)	11.15 (2/2)	86 (2/2)
16	IL (17.3%)	Vegetarian diet 6	29.75 (1/2)	24.79 (2/2)	6.58 (2/2)	85 (2/2)	7.11 (2/2)	85.5 (2/2)
17	IL (>1%)	Salad 2	34.48 (1/4)	34.45 (4/4)	9.28 (2/4)	85 (4/4)	10.05 (4/4)	86 (4/4)
18	IL (>1%)	Lasagna sauce	–	26.92 (4/4)	–	–	8.38 (4/4)	85.5 (4/4)
19	IL (>1%)	Vegetarian diet 7	33.92 (4/4)	29.16 (4/4)	9.17 (4/4)	85 (4/4)	8.67 (4/4)	85.5 (4/4)
20	IL (>1%)	Cookie 1	37.96 (4/4)	37.63 (4/4)	8.47 (4/4)	85.5 (4/4)	6.92 (4/4)	85.5 (4/4)
21	PAL	Endive mashed potato	38.58 (1/4)	37.96 (4/4)	–	–	8.92 (4/4)	85 (4/4)
22	PAL	Sauerkraut stew	–	38.40 (3/4)	–	–	9.73 (4/4)	85.5 (4/4)
23	PAL	Crackers	–	32.32 (4/4)	–	–	8.84 (4/4)	85.5 (4/4)
24	PAL	Veggie soup	–	38.31 (4/4)	–	–	8.17 (4/4)	85.5 (4/4)
25	PAL	Cheese spinach	–	31.37 (4/4)	9.85 (2/4)	85 (2/4)	8.55 (4/4)	85.5 (4/4)
26	PAL	Cookie 2	–	30.49 (4/4)	–	–	7.59 (4/4)	86 (4/4)
27	PAL	Bread	–	38.65 (2/4)	–	–	11.26 (3/4)	85.5 (3/4)
28	soy free	Salad 3	–	35.40 (4/4)	–	–	9.36 (4/4)	85.5 (4/4)
29	soy free	Cream cheese	–	40.29 (2/4)	–	–	9.42 (4/4)	85.5 (4/4)
30	soy free	Cheese sauce	–	37.45 (3/4)	–	–	13.17 (2/4)	85.5 (2/4)
31	soy free	Coco milk	–	42.59 (1/4)	9.79 (1/4)	85.5 (1/4)	17.58 (4/4)	86 (4/4)
32	not declared	Vegan herbs sauce	–	–	–	–	14.71 (4/4)	85 (4/4)

Note: [a] Ingredient labeling, IL (content), [b] Precautionary allergen labeling, PAL. [c] Time, average amplification time (The unit is minute). [d] Tm., average melting temperature (The unit is degrees C). [e] 25.66 (2/2) represents there were 2 positive reactions in 2 repeated tests in qPCR assay, and the average Cq value of the 2 positive results was 25.66 circles. [f] 5.55 (2/2) represents there were 2 positive reactions in 2 repeated tests in LAMP assay, and the average amplification time of the 2 positive results was 5.55 min. [g] 85 (2/2) represents there were 2 positive reactions in 2 repeated tests in LAMP assay, and the average melting temperature of 2 positive results was 85℃. - represents all negative results in the replicate tests.

Table S7. Comparison of different detection assays of soy allergen based on DNA technology.

Method	Food matrix	Sensitivity/LOD	Advantage	Disadvantage	Ref.
Recombinase polymerase amplification (RPA), PCR	Soybean seed	Sensitivity: 10 copies per reaction	· Fast DNA extraction and RPA procedure<br>· Duplex format detection with a reference gene and detecting gene<br>· Suitable for on-site detection	· Non representative samples with complex matrices<br>· Undeclared LOD	7
Post-PCR high resolution melting (HRM)	15 artificially prepared mixed samples of soy products of different matrices. 10 commercial spray creams were used for the application.	LOD: 8 copies of the soybean DNA	· Representative melting temperatures were validated<br>· Clear LOD determination	· Time-consuming<br>· Not applicable for on-site detection<br>· Limited representative commercial samples	8
Colorimetric LAMP microfluidic, qPCR	8 single-element plant samples for specificity test. 16 commercial food including 8 kinds of biscuits and candy, respectively, were tested for the application.	Sensitivity: detectable 0.4 ng/uL soybean DNA	· Three allergens were detected simultaneously by using a designed microfluidic chip<br>· Naked-eye visualization<br>· Suitable for on-site detection (after obtaining the extracted DNA)	· Limited representative commercial samples<br>· No reference gene<br>· Undeclared LOD	9
ELISA, PCR	57 food samples including 45 samples of animal origin and 12 samples of plant origin	No sensitivity exploration	· Four methods were evaluated on 57 foods and no significant difference between the tested methods	· No reference gene<br>· Undeclared LOD<br>· Time-consuming<br>· Not applicable for on-site detection	10
Body-heat recombinase polymerase amplification (RPA), electrophoresis	16 simple samples including soybean, rice, maize, potato, radish, beet etc.	No sensitivity exploration	· Lab instrument-free<br>· Portable naked-eye visualization using a mini-UV torch<br>· Made a contamination-prevention cartridge for RPA<br>· Suitable for on-site detection	· Non representative samples with complex matrices<br>· No reference gene<br>· Undeclared LOD	11
LAMP, electrophoresis	6 samples including drinks, candy, and biscuits.	Sensitivity: detectable 6.6 ng/μL of soybean DNA	· Fast DNA extraction based on glass fiber paper<br>· The entire DNA extraction and detection process within 1 h	· Non representative samples<br>· No reference gene<br>· No optimization of LAMP	12

				· Undeclared LOD<br>· Not applicable for on-site detection	
Digital droplet PCR	6 soybean samples and one maize flour were used for the validation. Food matrices including milk, pork meat, and a hazelnut creams for the application.	LOD: 0.16 mg/kg and LOQ 0.60 mg/kg	· The assay was validated using reference samples	· Non representative samples<br>· No reference gene<br>cross-reactivity rate<br>false-negative rates (37.5%)	13
Hybridization chain reaction coupled with gold nanoparticles	6 commercial food including biscuits and sweets	Sensitivity: detectable 0.5 nM of soybean DNA	· Naked eye distinguish (Positive result with light purple while negative result with red color)	· Non representative samples<br>· No reference gene<br>· No benchmark method<br>· Undeclared LOD (Did not mention how to define LOD)<br>· Not applicable for on-site detection	14
LAMP, LFD	35 simple food matrix (for example, almond, peanut, rye, pea, cow's milk, hen's egg, spelt, 100% tofu, etc) and 12 different soybean cultivars were tested.	No determination of LOD	· Explored the selection of soybean allergen genes<br>· LAMP product was interpreted by the LFD	· Non representative samples<br>· No reference gene<br>· No benchmark method for all samples<br>· Not applicable for on-site detection	5
LAMP, LFD, qPCR	19 single-element plant samples for specificity test. 12 retail foods were chosen for the application test. 8 retail food with various levels of processing were tested for the application.	No determination of LOD in pure soy DNA; 10 mg/kg in 3 incurred samples	· Evaluated the LAMP-LFD method by comparing two soy target genes<br>· Compared the sensitivity of LAMP-LFD and commercial protein-based LFD<br>· Less than 1 h (not including DNA extraction)	· No detail information about retail foods<br>· No reference gene<br>· Not applicable for on-site detection	15
qLAMP, LFD, qPCR	19 single-element plant samples for specificity test. 32 representative complex food samples containing macronutrients, four textures and different processing methods, were tested for the application.	In one LAMP reaction, 0.0005 ng and 0.005 ng of pure soy DNA and in 4 spiked samples	· Optimized the proposed method<br>· Reference genes for both LAMP and qPCR<br>· Duplex LFD test to ensure a reliable result<br>· Less than 20 min (not including DNA extraction)<br>· Traceable result by using a digital cube<br>· Clear LOD determination	· Suitable for on-site detection (after obtaining the extracted DNA)	Our work

Table S8. The semi quantitative results tested by LAMP-LFD assay equipped with digital cube and RFID-tag.

	Soy DNA (ng)	C line	T1 line (soy)	T2 line (cox)	Ratio: T1/C	RSD (%)	Ratio: T2/C	RSD (%)
test 1	5	113	120	122	1.06		1.08	
test 2	5	120	124	128	1.03	1.9	1.07	2.0
test 3	5	120	123	133	1.03		1.11	
test 1	5E-01	114	135	132.6	1.18		1.16	
test 2	5E-01	113	134	127	1.19	1.0	1.12	3.9
test 3	5E-01	115	134	139.7	1.17		1.21	
test 1	5E-02	120	128	132.6	1.07		1.11	
test 2	5E-02	119	124	133.8	1.04	1.0	1.12	2.0
test 3	5E-02	116	122	133.4	1.05		1.15	
test 1	5E-03	97	61	112	0.63		1.15	
test 2	5E-03	103	65	128	0.63	1.2	1.24	2.0
test 3	5E-03	107	64	108	0.60		1.01	
test 1	5E-04	119	0.00	3.10	0.00		0.03	
test 2	5E-04	112	0.00	0.00	0.00	0.0	0.00	0.0
test 3	5E-04	135	1.00	1.30	0.01		0.01	
test 1	5E-05	135	4.30	0.70	0.03		0.01	
test 2	5E-05	141	3.90	0.20	0.03	0.0	0.00	0.0
test 3	5E-05	140	4.70	0.20	0.03		0.00	

In this Table, different concentrations of pure soy DNA from 5 ng to 5 E-05 ng in LAMP reaction were tested. The readable and computable T1/C and T2/C data was interpreted as the detection results of the LAMP-LFD tests for soy and cox genes. The results here validate the detection capability for duplex LAMP-LFD using the digital cube, which is also consistent with the results depicted in Figure 2 in the main manuscript. This demonstrates that our developed proposal not only facilitates laboratory-based detection but also supports the reliability, reproducibility, and traceability of on-site detection through semi-quantitative assays.

References:

(1) Hird, H.; Chisholm, J.; Sanchez, A.; Hernandez, M.; Goodier, R.; Schneede, K.; Boltz, C.; Popping, B. Effect of Heat and Pressure Processing on DNA Fragmentation and Implications for the Detection of Meat Using a Real-Time Polymerase Chain Reaction. *Food Addit Contam* **2006**, *23* (7), 645–650. https://doi.org/10.1080/02652030600603041.

(2) Ross, G. M. S.; Filippini, D.; Nielen, M. W. F.; Salentijn, G. IJ. Unraveling the Hook Effect: A Comprehensive Study of High Antigen Concentration Effects in Sandwich Lateral Flow Immunoassays. *Anal Chem* **2020**, *92* (23), 15587–15595. https://doi.org/10.1021/acs.analchem.0c03740.

(3) Laube, I.; Hird, H.; Brodmann, P.; Ullmann, S.; Schöne-Michling, M.; Chisholm, J.; Broll, H. Development of Primer and Probe Sets for the Detection of Plant Species in Honey. *Food Chem* **2010**, *118* (4), 979–986. https://doi.org/10.1016/j.foodchem.2008.09.063.

(4) *European Union Reference Laboratory for GM Food and Feed (EU-RL-GMFF)*. http://gmo-crl.jrc.ec.europa.eu/[(accessed 2025-04-03).

(5) Allgöwer, S. M.; Hartmann, C. A.; Holzhauser, T. The Development of Highly Specific and Sensitive Primers for the Detection of Potentially Allergenic Soybean (Glycine Max) Using Loop-Mediated Isothermal Amplification Combined with Lateral Flow Dipstick (LAMP-LFD). *Foods* **2020**, *9* (4), 423. https://doi.org/10.3390/foods9040423.

(6) Tomlinson, J. A.; Dickinson, M. J.; Boonham, N. Rapid Detection of *Phytophthora Ramorum* and *P. Kernoviae* by Two-Minute DNA Extraction Followed by Isothermal Amplification and Amplicon Detection by Generic Lateral Flow Device. *Phytopathology* **2010**, *100* (2), 143–149. https://doi.org/10.1094/PHYTO-100-2-0143.

(7) Chandu, D.; Paul, S.; Parker, M.; Dudin, Y.; King-Sitzes, J.; Perez, T.; Mittanck, D. W.; Shah, M.; Glenn, K. C.; Piepenburg, O. Development of a Rapid Point-of-Use DNA Test for the Screening of Genuity® Roundup Ready 2 Yield® Soybean in Seed Samples. *Biomed Res Int* **2016**, *2016*, 1–12. https://doi.org/10.1155/2016/3145921.

(8) Sovová, T.; Křížová, B.; Kučera, L.; Ovesná, J. Detecting Soybean and Milk in Dairy and Soy Products with Post-PCR High Resolution Melting Assays. *Czech Journal of Food Sciences* **2020**, *38* (4), 209–214. https://doi.org/10.17221/125/2020-CJFS.

(9) Yuan, D.; Kong, J.; Li, X.; Fang, X.; Chen, Q. Colorimetric LAMP Microfluidic Chip for Detecting Three Allergens: Peanut, Sesame and Soybean. *Sci Rep* **2018**, *8* (1), 8682. https://doi.org/10.1038/s41598-018-26982-5.

(10) Kružíková, K. N.; Popelková, T.; Tšponová, Z.; Doubková, V.; Doleželová, P.; Laichmannová, L. Detection of Soy in Food from the Czech Market Using ELISA and PCR Methods. *Czech Journal of Food Sciences* **2022**, *40* (5), 375–382. https://doi.org/10.17221/100/2021-CJFS.

(11) Wang, R.; Zhang, F.; Wang, L.; Qian, W.; Qian, C.; Wu, J.; Ying, Y. Instant, Visual, and Instrument-Free Method for On-Site Screening of GTS 40-3-2 Soybean Based on Body-Heat Triggered Recombinase Polymerase Amplification. *Anal Chem* **2017**, *89* (8), 4413–4418. https://doi.org/10.1021/acs.analchem.7b00964.

(12) Sun, X.; Liu, Y.; Niu, B.; Chen, Q.; Fang, X. Rapid Identification and Quantitation of Single Plant Seed Allergen Using Paper-Based Microfluidics. *PLoS One* **2022**, *17* (12), e0266775. https://doi.org/10.1371/journal.pone.0266775.

(13) Mayer, W.; Schuller, M.; Viehauser, M. C.; Hochegger, R. Quantification of the Allergen Soy (Glycine Max) in Food Using Digital Droplet PCR (DdPCR). *European Food Research and*

Technology **2019**, *245* (2), 499–509. https://doi.org/10.1007/s00217-018-3182-5.

(14) Yuan, D.; Fang, X.; Liu, Y.; Kong, J.; Chen, Q. A Hybridization Chain Reaction Coupled with Gold Nanoparticles for Allergen Gene Detection in Peanut, Soybean and Sesame DNAs. *Analyst* **2019**, *144* (12), 3886–3891. https://doi.org/10.1039/C9AN00394K.

(15) Allgöwer, S. M.; Hartmann, C. A.; Lipinski, C.; Mahler, V.; Randow, S.; Völker, E.; Holzhauser, T. LAMP-LFD Based on Isothermal Amplification of Multicopy Gene ORF160b: Applicability for Highly Sensitive Low-Tech Screening of Allergenic Soybean (Glycine Max) in Food. *Foods* **2020**, *9* (12), 1741. https://doi.org/10.3390/foods9121741.
